# Mobile EEG identifies the re-allocation of attention during real-world activity

**DOI:** 10.1038/s41598-019-51996-y

**Published:** 2019-11-01

**Authors:** Simon Ladouce, David I. Donaldson, Paul A. Dudchenko, Magdalena Ietswaart

**Affiliations:** University of Stirling, Psychology, Faculty of Natural Sciences, Stirling, FK9 4LA United Kingdom

**Keywords:** Attention, Cognitive control

## Abstract

The distribution of attention between competing processing demands can have dramatic real-world consequences, however little is known about how limited attentional resources are distributed during real-world behaviour. Here we employ mobile EEG to characterise the allocation of attention across multiple sensory-cognitive processing demands during naturalistic movement. We used a neural marker of attention, the Event-Related Potential (ERP) P300 effect, to show that attention to targets is reduced when human participants walk compared to when they stand still. In a second experiment, we show that this reduction in attention is not caused by the act of walking *per se*. A third experiment identified the independent processing demands driving reduced attention to target stimuli during motion. ERP data reveals that the reduction in attention seen during walking reflects the linear and additive sum of the processing demands produced by visual and inertial stimulation. The mobile cognition approach used here shows how limited resources are precisely re-allocated according to the sensory processing demands that occur during real-world behaviour.

## Introduction

The distribution of attention between competing processing demands can have dramatic real-world consequences, as illustrated by the increased mortality risk associated with driving and simultaneous phone use^1,2^. Laboratory studies have long demonstrated that human attention has an inherently limited capacity^3,4^, but how limited attentional resources are distributed during real-world behaviour is largely unknown. Here, across three experiments, we employ the mobile cognition approach^5,6^ to show how limited resources are precisely re-allocated across competing sensory modalities when attention is distributed during real-world behaviour. More specifically, we utilise mobile electroencephalography (EEG) to characterise the re-allocation of attention that occurs in response to the multiple sensory-cognitive processing demands produced by naturalistic movement.

To measure attention, we assessed the Event-Related Potential (ERP) P300 effect^7,8^. The P300 effect is one of the most robust ERP components observed in human EEG recording. The traditional setting in which it is seen is the oddball paradigm, where participants are asked to pick out infrequently occurring stimuli (oddballs) from a context of alternative frequently occurring stimuli. In an auditory oddball task, for example, a participant may be asked to identify high-pitch target tones that occur infrequently within a series of repeated low-pitch tones. A large positive going deflection, known as the P300, is seen between 250 and 500 ms after infrequent tones, relative to repeated frequent tones. Concurrent neural imaging with fMRI suggests that the oddball P300 reflects variations in neural activity in the supramarginal gyri, thalamus, insula, and medial frontal gyrus^9^.

A fundamental property of the P300 effect is that its size decreases when attention is divided. For example, when participants are asked to pay attention to an auditory oddball while simultaneously performing a computer-based tracking task, the amplitude of the P300 associated with the oddball tones decreased^10^. This decrease in the size of the P300 effect during dual-task performance has been shown repeatedly^11^, and indicates that the amplitude of the P300 effect reflects the amount of attention allocated to detection of a target. For present purposes, in the context of questions about how attention is re-allocated during real-world behaviour, the auditory P300 effect is particularly relevant and suited to recording whilst participants perform other activities.

More broadly, the emergence of mobile brain imaging technology has allowed cognitive functions to be examined in increasingly complex real-world contexts. Such technology allows the recording of brain dynamics while participants are freely moving, including while engaged in natural behaviours in the face of an ever-changing environment. The mobile brain imaging approach allows questions to be asked that simply cannot be examined using laboratory experiments (e.g., the effects that changes in context have on episodic memory retrieval^12^), and has the potential to reveal embodied aspects of human cognitive experience that could not be captured within the framework of traditional laboratory settings^5,13^. However, applying this mobile brain imaging approach also brings methodological challenges related to data acquisition (reliability and consistency of mobile recordings), processing (disentangling brain signals from artifactual sources) and interpretation (integration of multimodal data collected in complex environments) that differ from the challenges inherent to traditional laboratory studies. Critically, the current studies demonstrate that such difficulties can be overcome.

Pioneering methodological developments using mobile EEG^14^ and functional Near-Infrared Spectroscopy (fNIRS)^15–17^ have validated mobile brain and body imaging methods. In relation to attention, previous mobile EEG studies have shown that neural correlates of attention are reduced during real-world behaviour including walking^18,19^, and cycling^20,21^, changes that cannot be accounted for by measurement-related artifacts associated with physical movement and must therefore be due to the changes in cognitive processing that occur during real-world behaviour. Other mobile fNIRS studies have shown an increase in oxygenation in frontal areas when participants were walking while performing a secondary task^22,23^. To date, however, the mobile cognition approach has not been used to identify the cognitive drivers underlying the re-allocation of attention during naturalistic real-world behaviour – which is the aim of the current work.

## Methods

The findings presented below come from three independent experiments (N = 11; 24; 24 respectively), all involving the recording of P300 ERP effects during performance of the same auditory oddball task (frequent and infrequent tones presented with a 4:1 ratio). Methodological characteristics that are shared across the experiments are presented here in the Methods section, with any additional distinctive information presented where relevant in the Results section of each experiment.

### Participants

All participants were healthy neurotypical young adults. All the participants were right-handed. Experimental protocols were approved by the University of Stirling ethics committee and informed consent was obtained from all participants. EEG procedures were carried out in accordance with standard guidelines^24^. Participants were excluded if the EEG data was affected by technical issues resulting in a failure to record sufficient data for analysis (i.e., due to cables becoming disconnected: five in Experiment 1, one in Experiment 2). Participants were also excluded if they did not follow the task instructions (operationalised as performance two standard deviations below the mean; one participant in Experiments 1 and 3).

### Stimuli and task

To record classic auditory oddball P300 ERP effects while participants were walking in a real-world environment we intentionally employed very distinct stimuli that could be easily discriminated from each other, and would allow high levels of performance (and therefore high trial numbers for EEG data analysis). All experiments employed the same two-tone auditory oddball paradigm, consisting of frequent high-pitch (1200 Hz) and infrequent low pitch (1000 Hz) tones, delivered under different conditions (e.g., standing and walking in Experiment 1, etc.). Individual participants completed all conditions within a single experiment; no participants took part in more than one Experiment; within each Experiment the condition order was counterbalanced across participants. A total of 300 tones were presented in each condition, presented in a pseudo-random order avoiding back-to-back targets. All tones were presented for 200 ms, with a fixed 800 ms inter-stimulus interval. The participants’ task throughout was to silently count infrequent low-pitch target tones, ignoring frequent high-pitch non-target tones.

All static conditions were completed in a single room with only the experimenter (SL) and participant present. Conditions involving traversal were completed at a natural pace, moving through University corridors with the participant followed (or pushed by) by the experimenter (SL), and involved occasional and incidental exposure to other people passing through the corridors. The hallway route was pre-planned by the experimenter and involved long straight sections with corners, but avoided stairs and the requirement to open doors. Prior to EEG data collection, participants went through a familiarization procedure to the route used in the experiment and also walked on a treadmill in order to define their normal walking pace. This subject-specific natural walking pace was then applied to experimental conditions involving treadmill walking (Experiment 2) but also to conditions including traversal of the environment in which the experimenter maintain the defined pace while pushing the participants in a wheelchair (Experiments 2 & 3). Where applicable, visual stimuli (pre-recorded videos of a grey wall or hallway traversal) were presented on a mobile phone screen held in a Google cardboard viewer that occluded peripheral vision of the surroundings, carefully fitted to the participant without interacting with EEG electrodes.

### EEG recording

**Figure 1:**
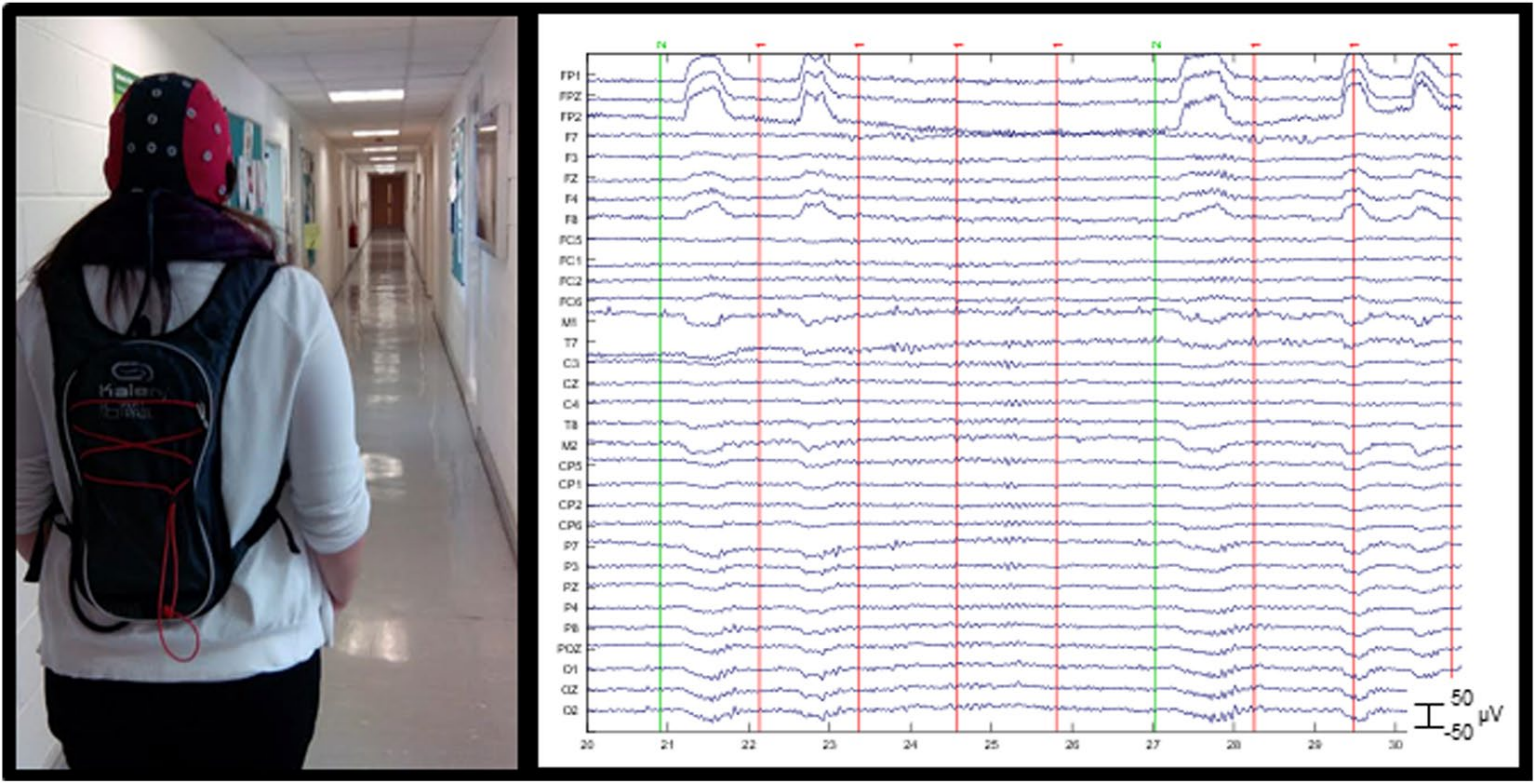
Mobile EEG: equipment, setup and recording. Left: Participant walking through a hallway of the University of Stirling while equipped with the mobile EEG system (the tablet and amplifier were fitted in a backpack). Right: Ten seconds of continuous EEG data recorded from 32 electrodes during performance on the auditory oddball task. Horizontal lines represent activity (in microvolts) recorded over time at each electrode. Vertical lines represent the onset of experimental events (target stimuli in green and non-target stimuli in red).

EEG data was recorded from 32 Ag/AgCl electrodes connected to a portable amplifier (ANT-neuro, Enschede, The Netherlands), with a sampling rate of 500 Hz and a 0.016–250 Hz bandpass filter. Electrodes (FP1, FPz, FP2, F7, F3, Fz, F4, F8, FC5, FC1, FC2, FC6, M1, T7, C3, Cz, C4, T8, M2, CP5, CP1, CP2, CP6, P7, P3, Pz, P4, P8, POz, O1, Oz, O2) were positioned according to the International 10–20 system^25^. Electrode AFz served as the ground and CPz as a common reference site. Electrode impedance was measured prior to each recording session and each channel was maintained below 5 kΩ using electrode gel. Stimulus presentation and timestamping of events was achieved using E-Prime (Version 2.0, Psychology Software Tools, Inc.) running on a PC laptop stored in the amplifier backpack (as shown in Fig. 1).

### Data processing

EEG data were analysed using the EEGLAB^26^ open source toolbox and custom MATLAB scripts (version R2014b 8.4.0.150421, The MathWorks Inc.). The continuous data was first visually examined and portions of the EEG displaying marked noise (including channel disconnections) were manually discarded. A Finite Impulse Response (FIR) band-pass filter ranging from 1 to 30 Hz was then applied to the continuous data (filter order: 16500, −6dB cut-off). Artifacts in the continuous EEG recording were identified and removed using extended infomax Independent Component Analysis (ICA^27^). Artifactual ICs were assessed based on their topographical distribution (pattern of signal activity across channels) and spectral features (power over different frequencies). ICs presenting prototypical features of common artifacts (e.g., associated with muscle movement, eye-blinks and heartbeat) or associated with signals presenting high variance and abnormal values were discarded manually following visual inspection. Moreover, source estimates of ICs obtained through dipole fitting were used as a complementary approach to confirm the selection and rejection of artifactual components. This approach resulted in the rejection of an average of 5 out of 32 (15%) ICs across all conditions of the three experiments reported (see Supplementary Information section for details).

Following ICA, EEG datasets were then epoched around the onset of auditory tones (−200 to 800 ms), aberrant epochs were excluded (using probability and kurtosis criteria of greater than two standard deviations from the mean) and baseline corrected (the mean voltage recorded within the 200 ms pre-stimulus period was subtracted from the signal for each electrode and each trial). The epoched data was then re-referenced to the average of the mastoids (M1 and M2) and average ERPs were formed according to stimulus type (frequent and infrequent) and condition (e.g., standing and walking in Experiment 1, etc.). Within each experiment the P300 component time window was defined empirically, across all conditions and all subjects (operationalised as two standard deviations around the mean single-trial peak latency between 250 and 500 ms). For each condition, the P300 effect was defined as the difference in amplitude between frequent and infrequent ERPs, at electrode Pz, averaged across the empirically defined condition-blind Experiment-specific time window. Mean amplitudes within the P300 time window were used for assessment of data quality (signal-to-noise ratios defined as P300 amplitude divided by the standard deviation of the pre-stimulus period) and statistical analyses (employing repeated measures ANOVA and paired-samples t-tests as appropriate to the experimental design). To ensure that parametric analysis was appropriate we first confirmed that all data followed a normal Gaussian distribution. In addition, for all post-hoc t-tests the Holm-Bonferroni correction for multiple comparisons was applied.

## Results

### Experiment 1

To assess how movement affects attention, mobile EEG was used to record the brain activity of participants (n = 11; 6 female, age range: 18–51; mean = 22) performing an auditory oddball paradigm, while either standing still or walking through a hallway. The oddball task involved binaural presentation of tones, played through USB speakers positioned on a lightweight, ergonomic backpack that also contained a wireless EEG amplifier. The participants’ task throughout was to silently count infrequent low-pitch target tones. Analysis of the behavioural data revealed that the difference between standing and walking was reflected in performance on the target detection task. Participants’ ability to detect targets was significantly lower during walking (2% and 4% target detection error rates during standing and walking respectively; t(10) = 2.319, p < 0.05, d = 0.699, BF_10_ = 1.920].

**Figure 2:**
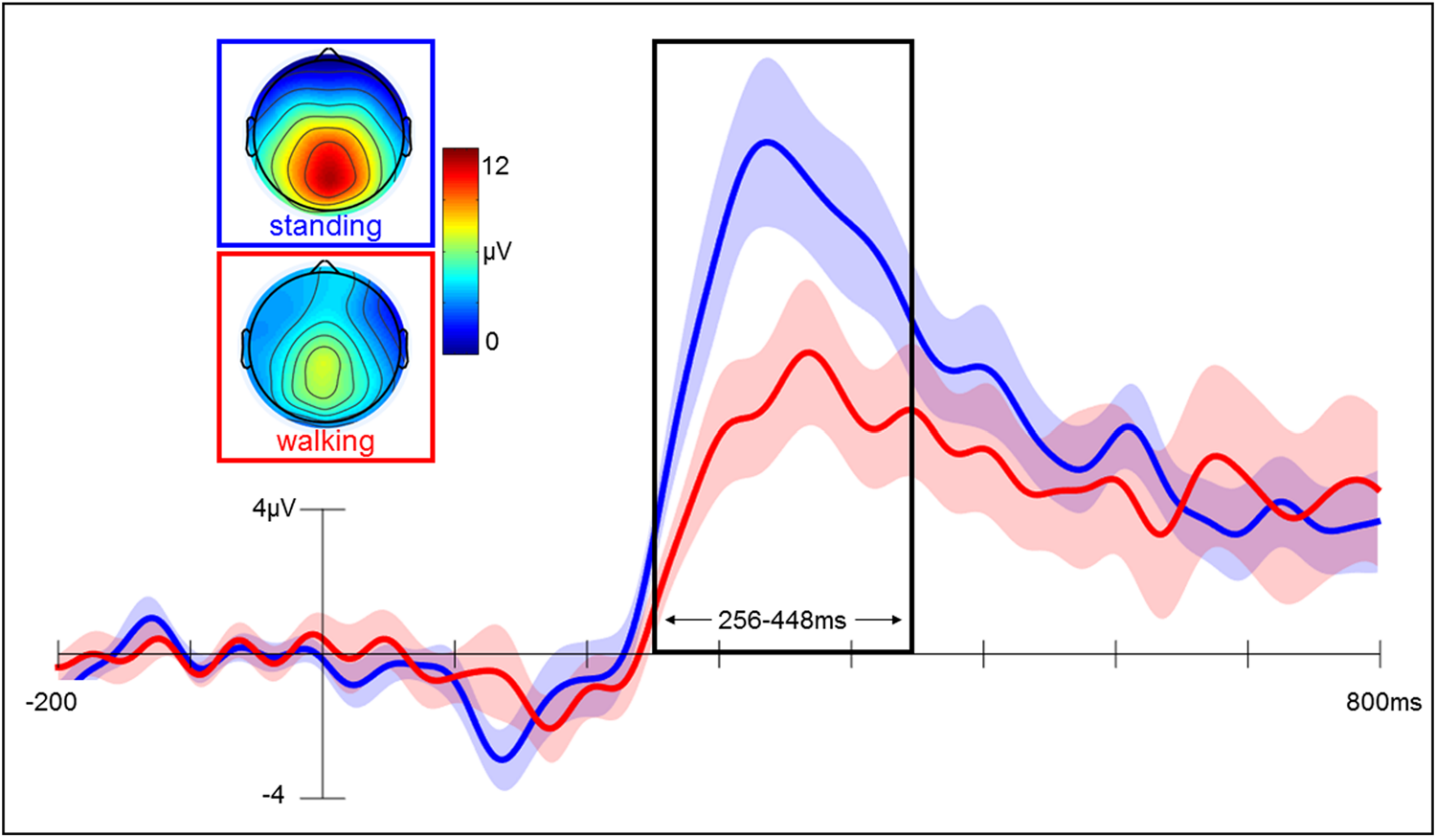
Experiment 1: The neural marker of attention is reduced during walking. Grand average (N = 11) difference waveforms (contrast between target and non-target elicited ERPs at electrode Pz) during standing (in blue) and walking (in red). Waveforms illustrate mean voltage (shaded area indicates standard error) derived from all available artifact free trials, with a 200 ms pre-stimulus baseline. Topographic maps illustrate the distribution of the magnitude of the P300 effect across the scalp, revealing clear midline parietal maxima for both standing and walking. The frame marked in black illustrates the data derived time window that defines the P300 effect (two standard deviations around the mean peak latency across all conditions: mean = 352 ms, SD = 47.8 ms), with no statistical difference in peak latency between standing (341 ms) and walking [363 ms; t(10) = 1.113, p = 0.292; d = 0.336, BF_10_ = 0.494].

EEG data were analysed to contrast ERPs time locked to target and non-target tones, revealing the P300 effect, a well characterised endogenous marker of cognitive processing associated with attention^7–10,28,29^. The ERP P300 difference waveforms from Experiment 1 are shown in Fig. 2, revealing that the magnitude of the P300 effect was significantly attenuated during walking (as shown by the difference within the black frame). While standing and walking both elicited statistically reliable P300 effects [t(10) = 6.322, p < 0.001, d = 1.906, BF_10_ = 325.52, and t(10) = 4.068, p < 0.01, d = 1.226, BF_10_ = 20.49, respectively], the P300 amplitude was significantly attenuated during walking [t(10) = 3.286, p < 0.01, d = 0.991, BF_10_ = 7.107].

Further details of the target and non-target ERPs used to form the ERP difference waveforms are reported in Supplemental Information. Here we provide evidence that the difference in the magnitude of the P300 effect does not simply reflect differences in data quality across conditions. First, as Fig. 2 shows, the neural data exhibits stable pre-stimulus baselines [i.e., assessed via the standard deviation of the baseline period: mean (s.d.) of 1.4 (0.51) and 1.5 (0.78) microvolts for standing and walking respectively]. Paired-sample t-tests confirmed that there were no differences in the variability of the data during the baseline period [t(10) = 0.337, p = 0.743, d = 0.102, BF_10_ = 20.49]. Second, the number of Independent Components rejected during data processing [mean (s.d.) of 3 (2) components for both standing and walking] did not differ across conditions [t(10) = 1.614, p = 0.138; d = 0.487, BF_10_ = 0.812]. Third, differences do not reflect variability in the numbers of trials contributing to the average waveforms. In all participants, a minimum of 25 out of 60 target trials contributed to the average waveforms (mean = 33, range from 25 to 44). Paired samples t-tests confirmed that there was no significant difference [t(10) = 0.529, p = 0.608] in the number of target trials recorded during standing (mean = 34; SD = 6) and walking (mean = 33; SD = 5). Similarly, there was no significant difference [t(10) = 0.729, p = 0.483] in the number of non-targets trials remaining for the standing (mean = 137; SD = 18) and walking (mean = 132; SD = 22) conditions.

In sum, although both the ERP and behavioural data suggest that walking led to a change in processing, the observed attenuation of the P300 effect during walking could simply be a consequence of having recorded brain activity during motion. Consequently, we conducted a second experiment in a larger number of participants to further investigate the reduction in magnitude of the P300 effect observed in Experiment 1.

### Experiment 2

A second experiment examined whether the act of walking per se explains the reduction of attention to target stimuli. Walking and standing place different demands on attention, for example in terms of the physical actions involved and the sensory inputs encountered. We examined these potential sources of attentional capture in a second group of participants (n = 24; 18 female, age range: 18–54, mean = 23.20), recording the P300 auditory oddball in the following conditions: 1) standing in an office, 2) sitting in a wheelchair that is pushed along a hallway, 3) walking on a treadmill, and 4) walking along a hallway. If the diminished P300 effect seen in Experiment 1 was due to the physical act of walking, it should be observed in both of the conditions that involve walking (walking and treadmill) compared to the non-walking conditions (standing and wheeled). By contrast, if the reduction in the P300 effect was due to the sensory input related to motion, then decreases should be observed in the two conditions involving traversal (walking or wheeled) compared to the stationary conditions (standing or treadmill). As we show below, our results support the latter view.

**Figure 3:**
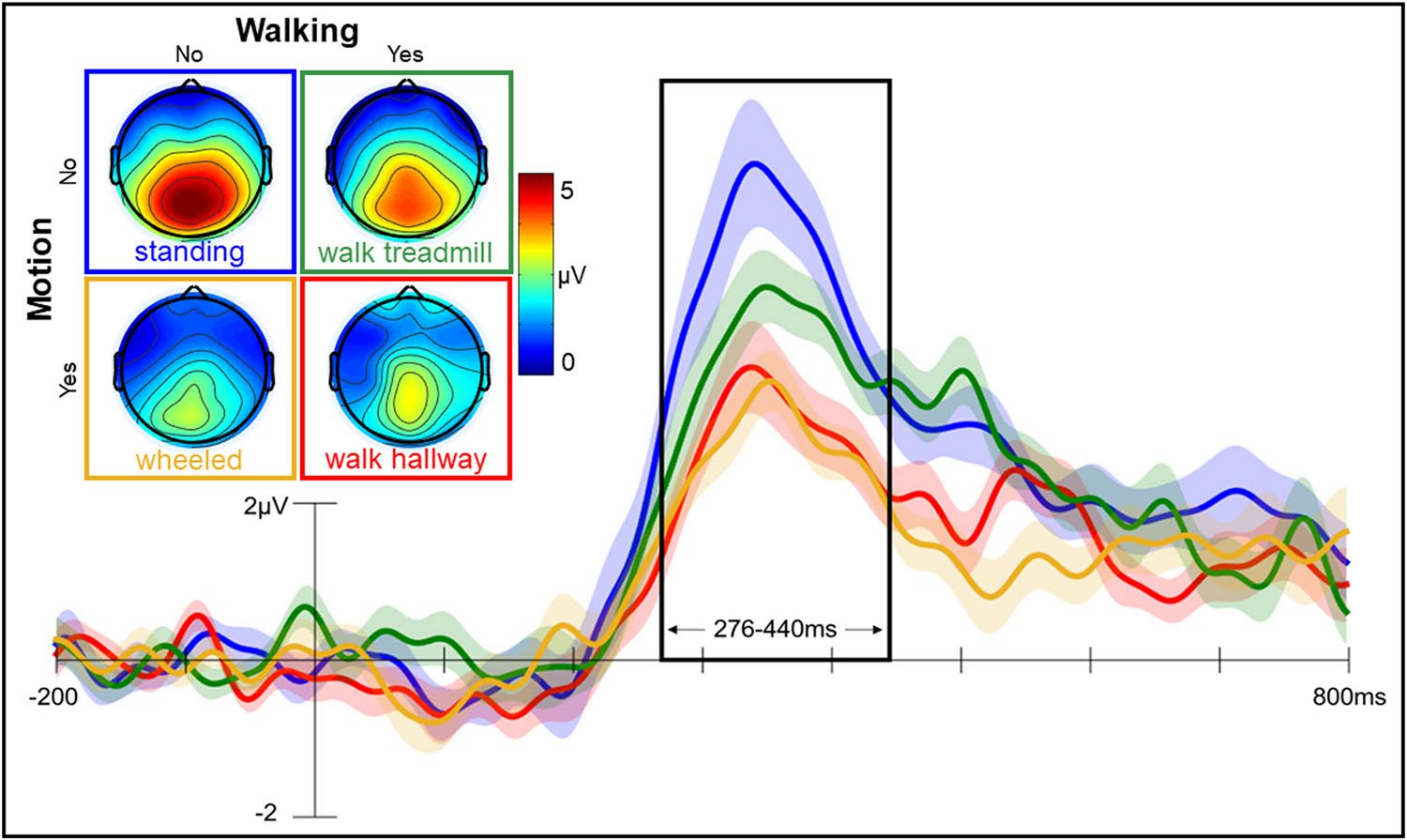
Experiment 2: Attention is not captured by walking per se. Grand average (N = 24) P300 difference waveforms during *standing* (in blue), walking on a *treadmill* (in green), walking through a *hallway* (in red) and being *wheeled* along a hallway (in yellow). Waveforms illustrate mean voltage (shaded area indicates standard error) derived from all available artifact free trials, with a 200 ms pre-stimulus baseline. Topographic maps illustrate the distribution of the P300 effect, revealing clear midline parietal maxima in all conditions. The black frame illustrates the data derived P300 time window (detailed in Supplementary Information).

The P300 effects from Experiment 2 are shown in Fig. 3, illustrating clear differences in the magnitude of effects across conditions. In all participants, at least 27 target trials contributed to the average waveforms (mean = 37, range from 27 to 60), and data quality is equivalent across conditions (see Supplementary Information for details). Given the design of Experiment 2 the magnitude of the P300 effect was analysed using repeated measures ANOVA with factors of motion (motion, no motion) and walking (walking, no walking), revealing a significant main effect of motion [F(1, 23) = 16.717, p < 0.001, ɳ^2^ = 0.421], no significant effect of walking [F(1, 23) = 2.329, p = 0.141, ɳ^2^ = 0.092], but a significant interaction between motion and walking [F(1, 23) = 6.434, p < 0.05, ɳ^2^ = 0.219]. Post-hoc contrasts confirmed that the P300 effect associated with attending to target tones in the hallway walking and the wheeled conditions was significantly smaller than that elicited when standing [walking: t(23) = 3.464, p < 0.01, d = 0.707, BF_10_ = 18.338; wheelchair: t(23) = 4.020, p < 0.001, d = 0.821, BF_10_ = 60.746]. Critically, the hallway walking and wheeled conditions produced equivalent modulations of attention [t(23) = 0.440, p = 0.664; d = 0.090, BF_10_ = 0.234]. Walking on a treadmill yielded a larger P300 effect than either the hallway walking [t(23) = 2.904, p < 0.01, d = 0.593, BF_10_ = 5.846] or the wheeled [t(23) = 3.289, p < 0.01, d = 0.671, BF_10_ = 12.729] conditions, but attention was still attenuated compared to when participants stood still [t(23) = 2.766, p < 0.05, d = 0.565, BF_10_ = 4.471].

Analysis of the behavioural data from Experiment 2 reveals that the motion-related reduction seen in the neural signal of attention is also evident in behavioural target-detection error-rates [F(1, 23) = 32.547, p < 0.001, ɳ^2^ = 0.586]. Follow up analysis (see Supplemental Information for details) confirmed that there was no significant difference in error rates between walking (4% error) and wheeled (4%) conditions, or between standing (2%) and treadmill (3%) conditions. The behavioural data therefore also rule out any potential concern that the environmental noise of steps on the treadmill or the noise of the wheels of the wheelchair might make it more difficult to discriminate the auditory target sounds.

In sum, Experiment 2 replicated the findings of Experiment 1. Participants once again showed a significantly reduced P300 ERP effect when walking through a hallway compared to when standing still. An equivalent reduction in the magnitude of the neural response was also observed when participants were wheeled along the hallway, suggesting that the availability of attention to targets was reduced because of the processing demands afforded by motion. By contrast, attention measured while walking on a treadmill approached that seen when standing still, ruling out the physical act of walking as the primary driver of reduced attention during walking. Given these findings, we carried out a final experiment to investigate which features of the processing demands generated by motion actually capture attention.

### Experiment 3

Taken together, the findings of Experiments 1 and 2 demonstrate that the reduction in attention to target stimuli seen during walking is not caused by the physical act of walking itself. Instead the marked reduction in the magnitude of the P300 effect that occurs during walking reflects a change in cognitive processing associated with the capture of attention. Although these findings provide clear evidence that processing associated with motion drives the re-allocation of attention, it remains unclear which features of the sensory experience of displacement capture attention. To address this question, a third group of participants (n = 24; 16 female, ages: 18–40, mean = 22.58) were tested on the auditory oddball paradigm as they: 1) sat facing a grey wall, 2) were wheeled along the hallway while watching a video of the grey wall, 3) sat stationary watching a video of traversal along the empty hallway, and 4) were wheeled along while freely viewing the hallway. If visual processing demands account for the re-allocation of attention seen during walking, then reduced P300 responses to targets should only occur in the conditions involving dynamic visual stimulation. By contrast, if processing of inertial stimulation accounts for the re-allocation of attention, then diminished P300 responses would occur in the conditions involving movement through space, and not in the stationary conditions. Critically, here the use of a factorial design allows us to ask whether the re-allocation of attention observed during motion is due solely to visual stimulation, purely to inertial stimulation, or some combination of the two.

**Figure 4:**
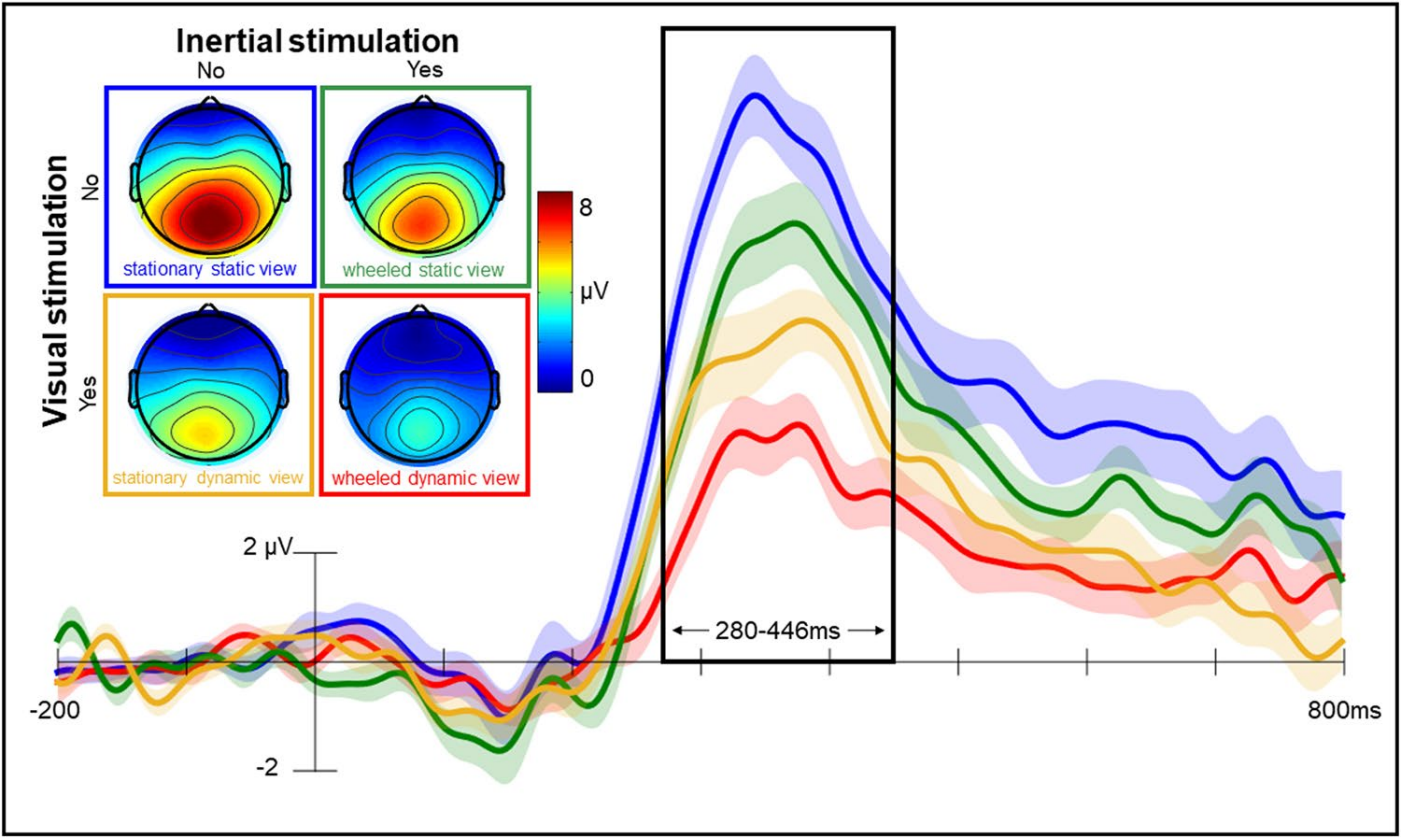
Experiment 3: Visual and inertial stimulation capture attention. Grand average (N = 24) P300 difference waveforms recorded while participants sat facing a grey wall (*stationary*, *static view* in blue), were wheeled along the hallway while watching a video of the grey wall, (*wheeled*, *static view* in green), sat stationary watching a video of traversal along the hallway (*stationary*, *dynamic view* in yellow) and were wheeled along the hallway while freely viewing the traversal (*wheeled*, *dynamic view* in red). Waveforms illustrate mean voltage (shaded area indicates standard error) derived from all available artifact free trials, with a 200 ms pre-stimulus baseline. Topographic maps illustrate clear midline parietal P300 maxima in all conditions. The black frame illustrates the data derived P300 time window (detailed in Supplementary Information).

The P300 effects from Experiment 3 are shown in Fig. 4, illustrating clear differences in the magnitude of effects across conditions. In all participants, at least 26 target trials contributed to the average waveforms (mean = 37, range from 26 to 51) and data quality is equivalent across conditions (see Supplementary Information for details). The magnitude of the P300 effect was analysed using repeated measures ANOVA with factors of visual and inertial stimulation, revealing significant main effects of both visual [F(1, 23) = 36.293, p < 0.001, ɳ^2^ = 0.612], and inertial [F(1, 23) = 23.387, p < 0.001, ɳ^2^ = 0.504] stimulation, but no interaction [F(1, 23) = 0.000, p = 0.998, ɳ^2^ = 0.000]. Inertial stimulation was associated with a 1.90 µV reduction in the size of the P300 effect, which was significantly larger [t(23) = 2.265, p < 0.05, d = 0.462, BF_10_ = 1.80] than the 3.28 µV reduction associated with visual stimulation. Critically, these two independent sources of stimulation account precisely for the 5.18 µV reduction in the size of the P300 effect seen when participants were wheeled along the hallway while freely viewing the traversal, demonstrating that together visual (63%) and inertial (37%) stimulation completely account for the capture of attention that occurs during motion.

The modulations observed in the neural signal of attention were also associated with changes in behavioural target detection error rates, with ANOVA revealing a significant main effect on task performance only for visual stimulation [F(1, 23) = 30.918, p < 0.001, ɳ^2^ = 0.573]. Conditions involving less visual stimulation produced significantly lower error rates: sat facing a grey wall (2%) and wheeled along the hallway while watching a video of the grey wall (3%), compared to sat watching a video of traversal along the hallway (5%) and wheeled along the hallway while freely viewing the traversal (5%). The equivalent error rates observed in the last two conditions is particularly notable because it once again rules out changes in physical noise (e.g., associated with movement of the wheels) as an explanation of the changes seen in the ERPs.

In sum, the findings of Experiment 3 demonstrate that visual and inertial stimulation contribute independently to the re-allocation of attention during motion. As shown in Fig. 4 the largest P300 effect is seen when there is neither visual nor inertial stimulation (i.e., when participants are performing the oddball task whilst sat facing a grey wall). By contrast, the biggest reduction in the P300 effect is seen when there is both visual and inertial stimulation (i.e., when wheeled along the hallway with free viewing of the traversal). Importantly, the data reveals dissociable effects of inertial and visual stimulation. Inertial stimulation alone is sufficient to produce a reduction in the P300 effect (i.e., when wheeled down the hallway viewing a video depicting a grey wall). Similarly, visual stimulation alone produces a reduction in the P300 effect (i.e., when stationary watching a video as if traversing along the hallway). Statistical comparison confirmed that visual stimulation produces a larger reduction in the magnitude of the P300 effect than inertial stimulation, leading to a significant impairment in performance on the primary target detection task. Remarkably, the reduction in size of the neural marker of attention seen when both visual and inertial stimulation are present reflects the linear and additive sum of the independent effects seen for visual and inertial stimulation alone.

## Discussion

Across three experiments we used mobile EEG to examine the cognitive drivers underlying the re-allocation of attention during naturalistic real-world behaviour. In an initial experiment we showed that attention allocated to the detection of infrequent target stimuli was reduced when human participants walk through a hallway compared to when they stand still. In a second experiment, we showed that this reduction in attention was not caused by the act of walking *per se*. Equivalent reductions in attention occurred for walking and being wheeled through the hallway, whereas attention measured while walking on a treadmill was comparable to that seen when standing still. In a third experiment we identified the independent processing demands driving reduced attention to target stimuli during motion: a significant reduction in attention occurred when participants travelled through the hallway with their vision obstructed (isolating the capture of attention by inertial stimulation), and a larger reduction when stationary but observing a video of hallway traversal (isolating attentional capture by visual stimulation). Taken together, these findings demonstrate that the reduction in attention seen during walking (compared to being stationary) reflects the linear and additive sum of the processing demands produced by visual and inertial stimulation.

The experiments presented here are not the first to demonstrate that mobile EEG can be used during real-world behaviour, nor are they the first to demonstrate reduction in the neural correlates of attention compared to traditional laboratory conditions. Indeed, previous studies have shown that neural correlates of attention are reduced during real-world behaviour including walking^18,19^, and cycling^20,21^. Importantly, however, the work presented here is the first to identify the specific sources of cognitive processing that drive changes in attention when target detection is carried out whilst walking around. Our data suggest that cognitive resources are precisely re-allocated according to the processing demands present during naturalistic behaviour. In the context examined here, the re-allocation of attentional resources was entirely attributable to two features of the sensory experience (the visual stimulation associated with displacement, and the inertial stimulation associated with movement) that have previously been shown to be key determinants of the firing of spatially tuned neurons in the rodent electrophysiological literature^30,31^. Overall, therefore, the present findings provide a clear cognitive explanation for why the neural correlates of attention are reduced during naturalistic behaviour, illustrating the modulation of attention by competing task demands.

The ability to precisely identify neural processing associated with changes in cognitive processing in the real world is important. As well as highlighting the opportunity to link findings in the animal literature with data in ‘freely moving’ humans (such as processes supporting navigation during motion) the present findings also have clear clinical and applied implications. To date, the static nature of brain imaging technology has largely focused human cognitive neuroscience towards modalities such as vision and hearing. By contrast, the mobile cognition approach used here highlights the role of inertial processing as a cognitive driver in real-world cognition. Animal work shows that vestibular information modulates hippocampal place cells, following the classic notion that sensory input is used to construct spatial maps required for basic spatial navigation^32,33^. By contrast, human vestibular function is relatively under-researched, although neuropsychological reports suggest that vestibular patients experience cognitive deficits in attention, learning and memory^34^. The impact of multisensory input on attentional processing during real-world behaviour identified here supports the idea that vestibular processes may serve as an embodied anchor for cognition^35^. In clinical contexts, therefore, quantification of the natural vestibular contribution to attentional disorders following brain damage may provide a better account of the difficulties experienced by patients in daily life, even after performance on traditional clinical assessments has returned to normal. The present findings suggest that ultimately it should be possible to quantify the cognitive demands placed by mobilisation in the elderly and neurological patients, providing a real-time measure of their remaining attentional capacity (and consequent ability to cope with additional demands).

The mobile cognition approach employed here brings a novel perspective to the study of human cognition, but also brings new technical challenges. In the current experiments, these challenges were experienced through the loss of event information or interruption of EEG recording, due to factors such as cable disconnections and the presence of pervasive noise in the EEG data (that precluded further analyses of some portions of data). From a methodological standpoint, these issues were tackled through the application of state-of the art signal processing procedures and the rejection of data exhibiting artefacts to increase signal-to-noise ratios. As a consequence, a substantial number of trials (over 40%) had to be excluded across the three experiments. Indeed, several datasets had to be completely discarded due to technical issues and initial difficulties with reliable data collection (resulting in a smaller sample size for our first experiment in particular). It is therefore important to consider these practical issues when planning experiments involving the recording of brain imaging data during real-world behaviour. Although novel solutions are being developed (e.g., automatic classification of artifactual components in the EEG^36^; dual-electrodes motion artifact cancellation^37^) that have the potential to improve mobile EEG data acquisition and processing, at present it remains sensible to account for a larger margin of data loss in real-world studies than is expected in traditional laboratory studies. Regardless, one important feature of the current findings is that the variation in the magnitude of the P300 effect we observed does not reflect variability in data quality, as reflected by analysis of baseline variability, signal to noise properties and artefact rejection rates (see Supplemental information for details). Instead, our findings very specifically identify cognitive processing demands that drive the re-allocation of attention during real-world behaviour.

Another challenge inherent to the collection of EEG data in the real-world (in comparison to laboratory settings) is a relative loss of experimental control. Across our experiments, several conditions involved walking or being wheeled through corridors. During these conditions the perceptual experience necessarily varies, as features of the environment change. For example, the “wheeled dynamic view” condition very occasionally included human encounters that could have momentarily affected attention, whereas the “stationary dynamic view” presented a recording of a dynamic environment that did not include any exposure to other individuals. Clearly, the presence of natural variation between conditions that are theoretically matched (e.g., in terms of perceptual experience) potentially introduces confounds that reduce the internal validity of the experimental design. Our view, backed up by the present findings, is that the disadvantages of this loss of control are more than offset by the advantages associated with the ability to examine behaviour in real-world contexts. More broadly, the present findings highlight the fact that the design of real-world studies poses new conceptual challenges that will require careful consideration, and some compromise, if both experimental control and ecological validity are to be served.

It is worth noting that mobile brain imaging methods and the field of mobile cognition are still very much in their infancy and undergoing rapid development. Scientific effort to date has largely focused on addressing the challenges and limitations of mobile EEG, with particular emphasis on technical innovation. For example, dry electrode sensor systems^38^ have been shown to enhance flexibility and ease of use during capping, making mobile technologies more suited to assessments in everyday settings^39^. Similarly, proof-of-concept studies have demonstrated the viability of simplified electrode arrays, such as around-the-ear^40,41^ and intra-auricular^42^ EEG sensors. One advantage of these new EEG systems is their small physical footprint and inconspicuous appearance, which should reduce the self-consciousness associated with wearing EEG arrays in public. These technological advances are important at least in part because they enhance the ecological validity of studies designed to characterize natural behaviours in real world contexts (e.g., sports, ergonomics), particularly those involving the study of human interactions^5^. Alongside hardware improvements, novel signal processing methods have been developed to address the unique challenges posed by the recording of brain dynamics in freely moving individuals^43,44^. Notably, recent efforts have focused on the use of phantom-head electrodes for the modelling of motion-related noise^37^, providing tailored solutions for the estimation of source locations based on signals acquired through around-the-ear and intra-auricular EEG arrays^45^.

Rather than focus on technological development, our aim was to show that mobile EEG can be used to enhance our understanding of cognitive processes. By examining the neural response to experimentally presented target stimuli when additional demands were present, our findings demonstrate that a known neural marker can be used to identify changes in attention during real-world behaviour. In principle, however, we might also have expected to be able to observe changes in neural activity associated with additional processing (i.e., related to visual and inertial stimulation). For example, attention to information in different modalities should give rise to changes in the network of regions recruited, which could be revealed via connectivity or coherence analysis. To our minds, however, identifying the neural correlates of attention to visual and inertial processing would require a different experimental paradigm. More specifically, neural activity would need to be examined in relation to the visual and inertial processing demands, rather than in relation to the presentation of auditory target stimuli (as is done here). In practice, the current experiments did not include any specific information about the features of the on-going stream of visual and inertial stimulation being processed, or exactly when such processing occurred. For this reason, our analysis solely focussed on observing changes in the processing of target stimuli, which allowed us to reveal that some of the attention allocated to processing these stimuli when stationary was no longer available when the same stimuli where attended to during walking. An important aim for future studies is to identify distinct neural markers associated with processing different types of information, to examine whether linear and additive reductions in attention to one source are matched by a concomitant linear and additive increase in attention to another.

Clearly, in contexts other than the present study, the specific sources of information capturing attention will vary depending on the task goals and environmental conditions. Whatever those particular demands are, the present findings suggest that the overall effect on attention is likely to reflect the linear sum of all stimulation sources. One striking implication of these findings is that attentional capture can be identified and measured in real-world contexts where reductions in attention really matter. For example, mobile EEG technology allows attentional capture to be monitored during simple but potentially dangerous activities like crossing the road, as well as during complex activities such as driving a car^46^ or even piloting an aircraft^47^. Importantly, our findings highlight the possibility of quantifying the relative contribution of different sources of attentional capture in more complex real-world contexts, such as air-traffic control or tram driving, where identifying and removing detrimental sources of stimulation may make the difference between life and death.

## Supplementary information

Supplementary Information

## Data Availability

The data supporting the findings of the work are available in the DataSTORRE repository: http://hdl.handle.net/11667/137.

## References

[1] Caird JK, Johnston KA, Willness CR, Asbridge M, Steel P (2014). A meta-analysis of the effects of texting and driving. Accident Analysis and Prevention.

[2] Wilson FA, Stimpson JP (2010). Trends in fatalities from distracted driving in the United States, 1999 to 2008. American Journal of Public Health.

[3] Broadbent, D. *Perception and Communication*. London: Pergamon Press (1958).

[4] Kahneman, D. *Attention and effort*. New Jersey: Prentice-Hall Inc (1973).

[5] Ladouce S, Donaldson DI, Dudchenko PA, Ietswaart M (2017). Understanding Minds in Real-World Environments: Toward a Mobile Cognition Approach. Frontiers in Human Neuroscience.

[6] Park, J. L., Dudchenko, P. A., & Donaldson, D. I. Navigation in real-world environments: new opportunities afforded by advances in mobile brain imaging. *Frontiers in Human Neuroscience* **12** (2018) 10.3389/fnhum.2018.00361 PMC6141718 30254578

[7] Näätänen R (1990). The role of attention in auditory information processing as revealed by event-related potentials and other brain measures of cognitive function. Behavioral and Brain Sciences.

[8] Polich J (2007). Updating P300: An Integrative Theory of P3a and P3b. Clinical Neurophysiology.

[9] Horowitz SG, Skudlarski P, Gore JC (2002). Correlations and dissociations between BOLD signal and P300 amplitude in an auditory oddball task: a parametric approach combining fMRI and ERP. Magnetic Resonance Imaging.

[10] Wickens C, Kramer A, Vanasse L, Donchin E (1983). Performance of concurrent tasks: A psychophysiological analysis of the reciprocity of information-processing resources. Science.

[11] Näätänen, R. *Attention and Brain Function*. Lawrence Erlbaum Associates, Hove and London (1992).

[12] Park JL, Donaldson DI (2019). Detecting the neural correlates of episodic memory with mobile EEG: Recollecting objects in the real world. NeuroImage.

[13] Gramann K (2011). Cognition in action: Imaging brain/body dynamics in mobile humans. Reviews in the Neurosciences.

[14] Reis PMR, Hebenstreit F, Gabsteiger F, von Tscharner V, Lochmann M (2014). Methodological aspects of EEG and body dynamics measurements during motion. *Frontiers in Human*. Neuroscience.

[15] Piper SK (2014). A Wearable Multi-Channel fNIRS System for Brain Imaging in Freely Moving Subjects. NeuroImage.

[16] Pinti P (2015). Using Fiberless, Wearable fNIRS to Monitor Brain Activity in Real-world Cognitive Tasks. Journal of Visualized Experiments.

[17] Quaresima, V. & Ferrari, M. Functional Near-Infrared Spectroscopy (fNIRS) for Assessing Cerebral Cortex Function During Human Behavior in Natural/Social Situations: A Concise Review. Organizational Research Methods, 1–23 (2016).

[18] Debener S, Minow F, Emkes R, Gandras K, de Vos M (2012). How about taking a low-cost, small, and wireless EEG for a walk?. Psychophysiology.

[19] De Vos M, Gandras K, Debener S (2014). Towards a truly mobile auditory brain-computer interface: Exploring the P300 to take away. International Journal of Psychophysiology.

[20] Zink R, Hunyadi B, Van Huffel S, De Vos M (2016). Mobile EEG on the bike: disentangling attentional and physical contributions to auditory attention tasks. Journal of Neural Engineering.

[21] Scanlon, J. E. M., Sieben, A. J., Holyk, K. R., & Mathewson, K. E. Your brain on bikes: P3, MMN/N2b, and baseline noise while pedalling a stationary bike. *Psychophysiology* **00** (2017) 10.1111/psyp.12850 28247405

[22] Holtzer R (2015). Online fronto-cortical control of simple and attention-demanding locomotion in humans. NeuroImage.

[23] Mirelman A (2014). Increased frontal brain activation during walking while dual tasking: an fNIRS study in healthy young adults. J. Neuroeng. Rehabil..

[24] Picton TW (2000). Guidelines for using human event-related potentials to study cognition: Recording standards and publication criteria. Psychophysiology.

[25] Jasper HH (1958). Report of the committee on methods of clinical examination in electroencephalography. Electroencephalography and Clinical Neurophysiology Supplement.

[26] Delorme A, Makeig S (2004). EEGLAB: an open source toolbox for analysis of single-trial EEG dynamics including independent component analysis. Journal of Neuroscience Methods.

[27] Bell AJ, Sejnowski TJ (1995). Information-Maximization Approach to Blind Separation and Blind Deconvolution. Neural Computation.

[28] Sutton S, Braren M, Zubin J (1965). Evoked-Potential Correlates of Stimulus Uncertainty. Science.

[29] Isreal JB, Chesney GL, Wickens CD, Donchin E (1980). P300 and Tracking Difficulty: Evidence For Multiple Resources in Dual-Task Performance. Psychophysiology.

[30] Harvey CD, Collman F, Dombect DA, Tank DW (2009). Intracellular dynamics of hippocampal place cells during virtual navigation. Nature.

[31] Chen G, King JA, Burgess N, O’Keefe J (2013). How vision and movement combine in the hippocampal place code. Proceedings of the National Academy of Sciences.

[32] Dix MR, Hallpike CS (1952). The pathology, symptomatology and diagnosis of certain common disorders of the vestibular system. Annals of Otology, Rhinology & Laryngology.

[33] Keefe JO, Burgess N, Donnett JG, Je KJ, Maguire EA (1998). Place cells, navigational accuracy, and the human hippocampus. Philosophical Transactions of the Royal Society B: Biological Sciences.

[34] Smith PF, Zheng Y, Horii A, Darlington CL (2005). Does vestibular damage cause cognitive dysfunction in humans?. Journal of Vestibular Research: Equilibrium & Orientation.

[35] Ferrè ER, Lopez C, Haggard P (2014). Anchoring the Self to the Body: Vestibular Contribution to the Sense of Self. Psychological Science.

[36] Pion-Tonachini L, Kreutz-Delgado K, Makeig S (2019). ICLabel: An automated electroencephalographic independent component classifier, dataset, and website. Neuroimage.

[37] Nordin AD, Hairston WD, Ferris DP (2018). Dual-electrode motion artifact cancellation for mobile electroencephalography. Journal of Neural Engineering.

[38] Lopez-Gordo M, Daniel Sanchez-Morillo, Valle F (2014). Dry EEG electrodes. Sensors.

[39] Arico, P., Borghini, G., Di Flumeri, G., Sciaraffa, N. & Babiloni, F. Passive BCI beyond the lab: Current trends and future directions. Physiological Measurement (2018). 10.1088/1361-6579/aad57e 30039806

[40] Debener S, Emkes R, De Vos M, Bleichner M (2015). Unobtrusive ambulatory EEG using a smartphone and flexible printed electrodes around the ear. Sci. Rep..

[41] Pacharra M, Debener S, Wascher E (2017). Concealed Around-the-Ear EEG Captures Cognitive Processing in a Visual Simon Task. Frontiers in Human Neuroscience.

[42] Mikkelsen KB, Kappel SL, Mandic DP, Kidmose P (2015). EEG recorded from the ear: Characterizing the Ear-EEG Method. Frontiers in Neuroscience.

[43] Minguillon, J., Lopez-Gordo, M. A. & Pelayo, F. Trends in EEG-BCI for daily-life: Requirements for artifact removal. *Biomedical Signal Processing and Control* (2017)

[44] Mihajlovic V, Grundlehner B, Vullers R, Penders J (2015). Wearable, wireless EEG solutions in daily life applications: What are we missing?. IEEE J. Biomed. Heal. Informatics.

[45] Kappel SL, Makeig S, Kidmose P (2019). Ear-EEG Forward Models: Improved Head-Models for Ear-EEG. Frontiers in Neuroscience.

[46] Protzak J, Gramann K (2018). Investigating Established EEG Parameter During Real-World Driving. Frontiers in Psychology.

[47] Borghini G, Astolfi L, Vecchiato G, Mattia D, Babiloni F (2014). Measuring neurophysiological signals in aircraft pilots and car drivers for the assessment of mental workload, fatigue and drowsiness. Neuroscience and Biobehavioral Reviews.

