# Supplementary material for: Mobile EEG identifies the re-allocation of attention during real-world activity
Source: Sci Rep. 2019 Nov 1;9:15851. doi: 10.1038/s41598-019-51996-y (PMC6825178; doi:10.1038/s41598-019-51996-y)
Supplement: Supplementary file 1 — Supplementary Information [file 41598_2019_51996_MOESM1_ESM.pdf]

**Mobile EEG identifies the re-allocation of attention during real-world activity.**

Simon Ladouce[1]*, David I. Donaldson[1], Paul A. Dudchenko[1], and Magdalena Ietswaart[1]

*Correspondence to simon.ladouce@stir.ac.uk

[1] University of Stirling

Psychology, Faculty of Natural Sciences

Stirling, FK9 4LA

United Kingdom

Phone: +44 1786 466 857

**Supplementary Information**

***Experiment 1***

Supplementary Figures 1 (standing) and 2 (walking) illustrate the ERP waveforms for frequent and infrequent tones recorded in Experiment 1, highlighting that the ERPs recorded during standing and walking both exhibit robust P300 effects, despite clear differences in the morphology of the raw ERP waveforms.

Given that the experimental design required ERPs to be compared across standing and walking conditions it is important to demonstrate that any differences in the magnitude of the P300 effect are not simply an artifact produced by differences in noise. In the main text we reported that the number of trials remaining after artifact rejection was not significantly affected by the recording condition. In addition, here we also confirm that there was no statistical difference [$t(10) = 1.614$, $p = .138$] in the number of Independent Components (ICs) identified as artifacts during data processing between standing (mean = 3; SD = 2) and walking (mean = 3; SD = 2) conditions. Taken together, the data quality assessment strongly suggests that the observed differences in the size of the P300 effect cannot be explained by artifacts.

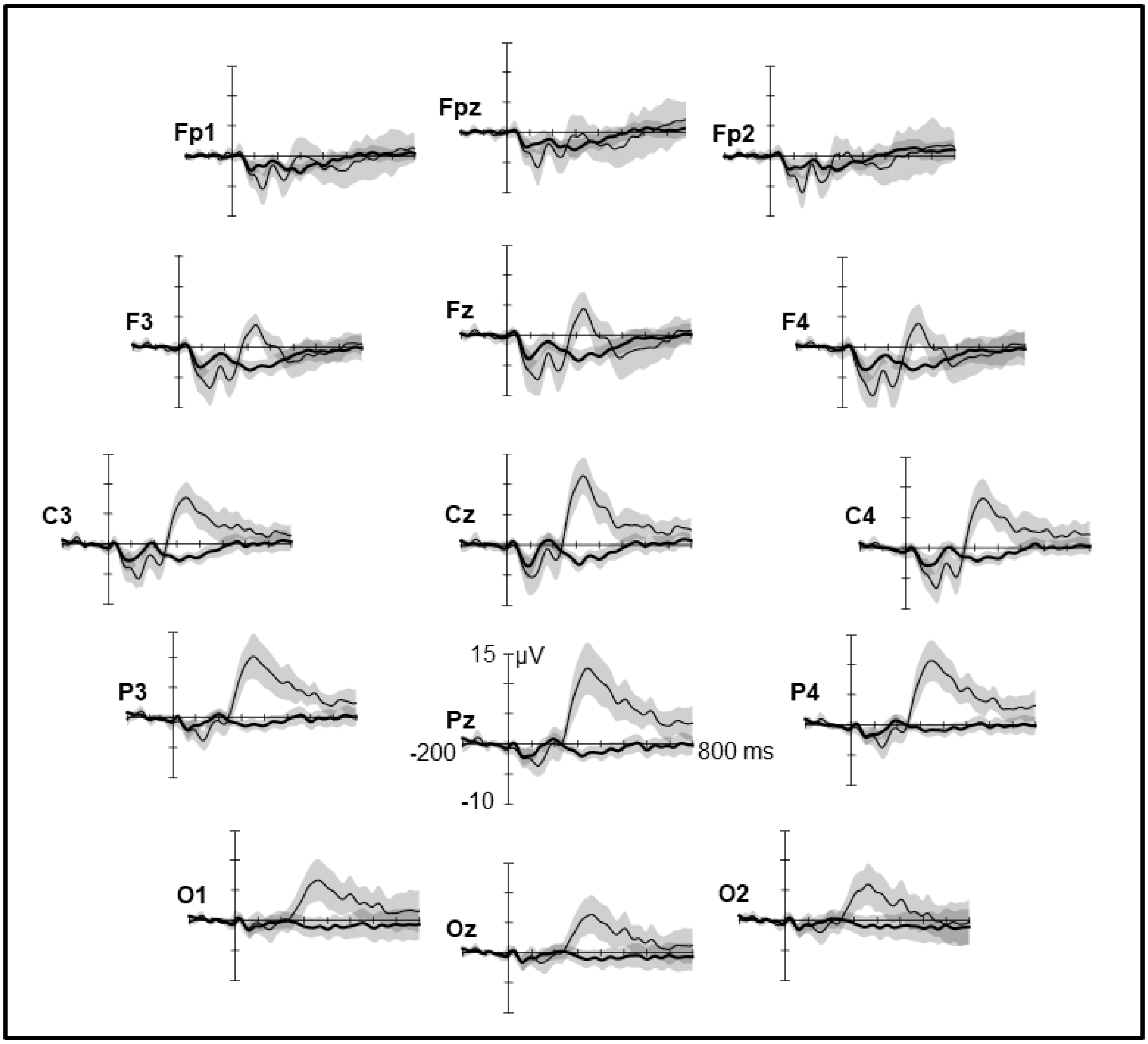


**Supplementary Figure 1: Experiment 1 - Standing.** Distribution of grand-average (N=11) ERP waveforms at midline and medial electrode sites for the frequent (thick line) and infrequent (thin line) tones, recorded as participants performed the auditory oddball task while standing. Data show mean voltage and associated standard error (shaded area), with a 200ms pre-stimulus baseline period. These data were used to generate the P300 ERP difference waveforms shown in Figure 2.

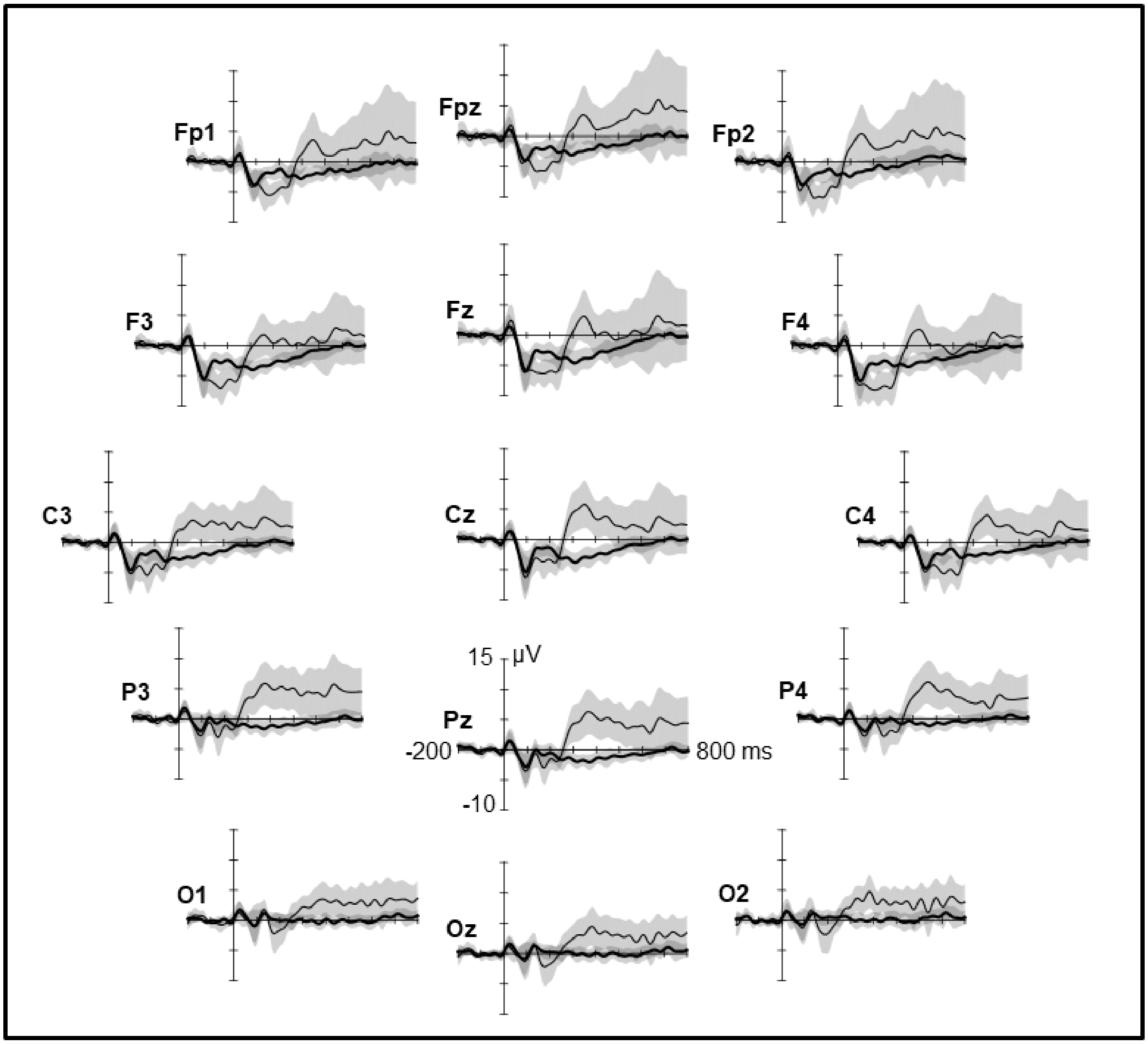


**Supplementary Figure 2: Experiment 1 - Walking.** Distribution of grand-average (N=11) ERP waveforms at midline and medial electrode sites for the frequent (thick line) and infrequent (thin line) tones, recorded as participants performed the auditory oddball task while walking. These data are shown as in Supplementary Figure 1, and were used to generate the P300 ERP difference waveforms shown in Figure 2.

Since the primary goal of the first study was to assess the classic auditory oddball P300 effect in the real-world while participants were walking, stimuli were chosen that could be easily discriminated from each other during motion in a potentially noisy environment. Unsurprisingly, therefore, participants correctly reported 98% (SD = 1) of targets stimuli presented in the standing condition and 96% (SD = 2) of targets that were presented in the walking condition. This near-perfect task performance was observed across all participants, suggesting that the tones presented were consistently audible, despite the potential presence of external noise sources during the walking condition. As noted in the main text task performance was significantly higher for the standing condition than in the walking condition [$t(10) = 2.319$, $p < .05$, $d = .699$, $BF_{10} = 1.920$]. Analysis also revealed marginally significant correlations between P300 amplitude and task performance across conditions [standing, $r_{10} = .492$, $p = .124$, two-tailed; walking, $r_{10} = .595$, $p = .054$, two-tailed], most likely reflecting a ceiling effect due to the task being designed to make the perceptual discrimination easy to perform.

***Experiment 2***

Supplementary Figures 3 to 6 illustrate the ERP waveforms for frequent and infrequent tones recorded in Experiment 2, from which the P300 difference waveforms reported in the main text were formed. In the main text we reported comprehensive analysis of data quality for Experiment 1; here we provide equivalent information for the data reported in Experiment 2. The ERP data were derived from all available artifact free trials; mean of 37 targets (range: 27-66) and 156 non-targets (range: 118-248) across conditions and

participants. For targets the means (and SDs) were: 37 (5) for standing, 38 (4) for treadmill, 37 (5) for walking through a hallway and 37 (7) for being wheeled along a hallway. For non-targets the means (and SDs) were: 154 (16) for standing, 160 (16) for treadmill, 158 (25) for walking through a hallway and 154 (26) for being wheeled along a hallway. As in Experiment 1, the number of trials remaining after artifact rejection was not significantly affected by recording condition. To compare trials across conditions we employed ANOVA with the factors of motion and walking; analysis revealed no significant effect of walking on the amount of non-target [$F(1,23) = 1.175$, $p = .290$], or target [$F(1,23) = .519$, $p = .478$] trials remaining after artifact rejection. Similarly, analysis revealed no significant effect of motion on the amount of non-target [$F(1,23) = .064$, $p = .803$] or target [$F(1,23) = .200$, $p = .659$] trials remaining. Moreover, no interaction was found between motion and walking for either non-target [$F(1,23) = .027$, $p = .871$] or target [$F(1,23) = .214$, $p = .648$] trials remaining.

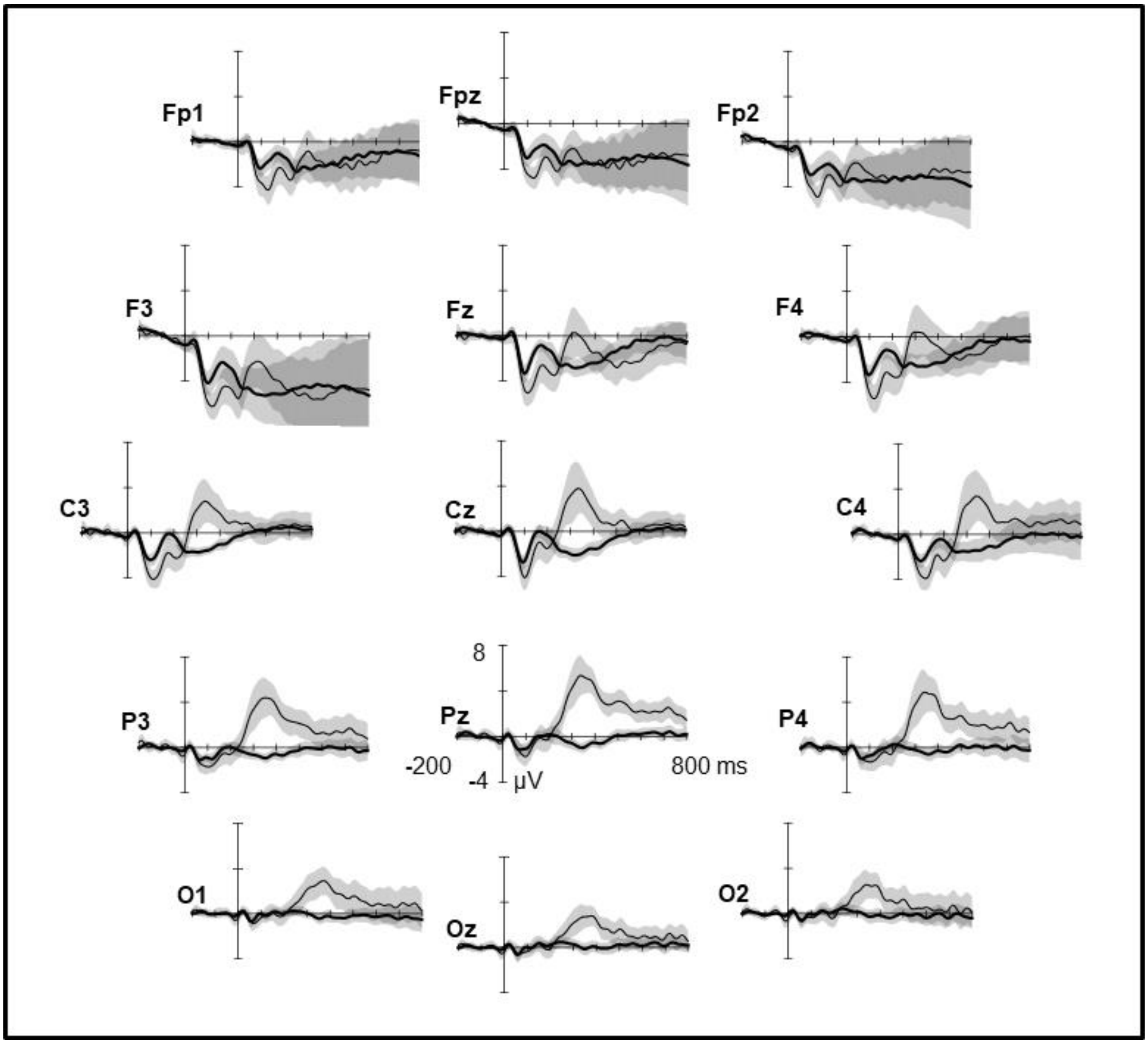


**Supplementary Figure 3: Experiment 2 - Standing.** Distribution of grand-average (N=24) ERP waveforms at midline and medial electrode sites for the frequent (thick line) and infrequent (thin line) tones, recorded as participants performed the auditory oddball task while standing. These data are shown as in Supplementary Figure 1 and were used to generate the P300 ERP difference waveforms shown in Figure 3.

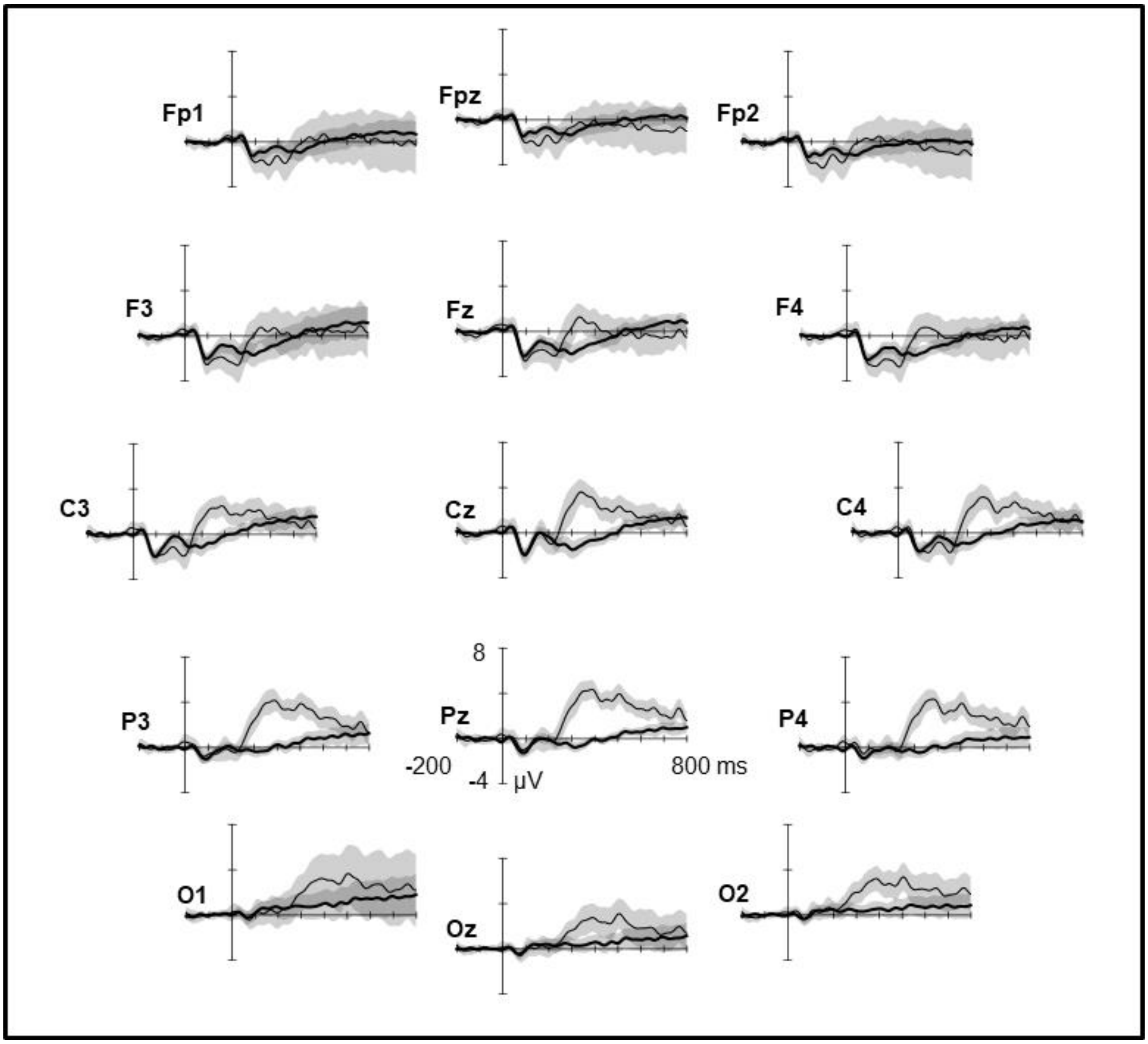


**Supplementary Figure 4: Experiment 2 - Walk treadmill.** Distribution of grand-average (N=24) ERP waveforms at midline and medial electrode sites for the frequent (thick line) and infrequent (thin line) tones, recorded as participants performed the auditory oddball task while walking on a treadmill. These data are shown as in Supplementary Figure 1 and used to generate the P300 ERP difference waveforms shown in Figure 3.

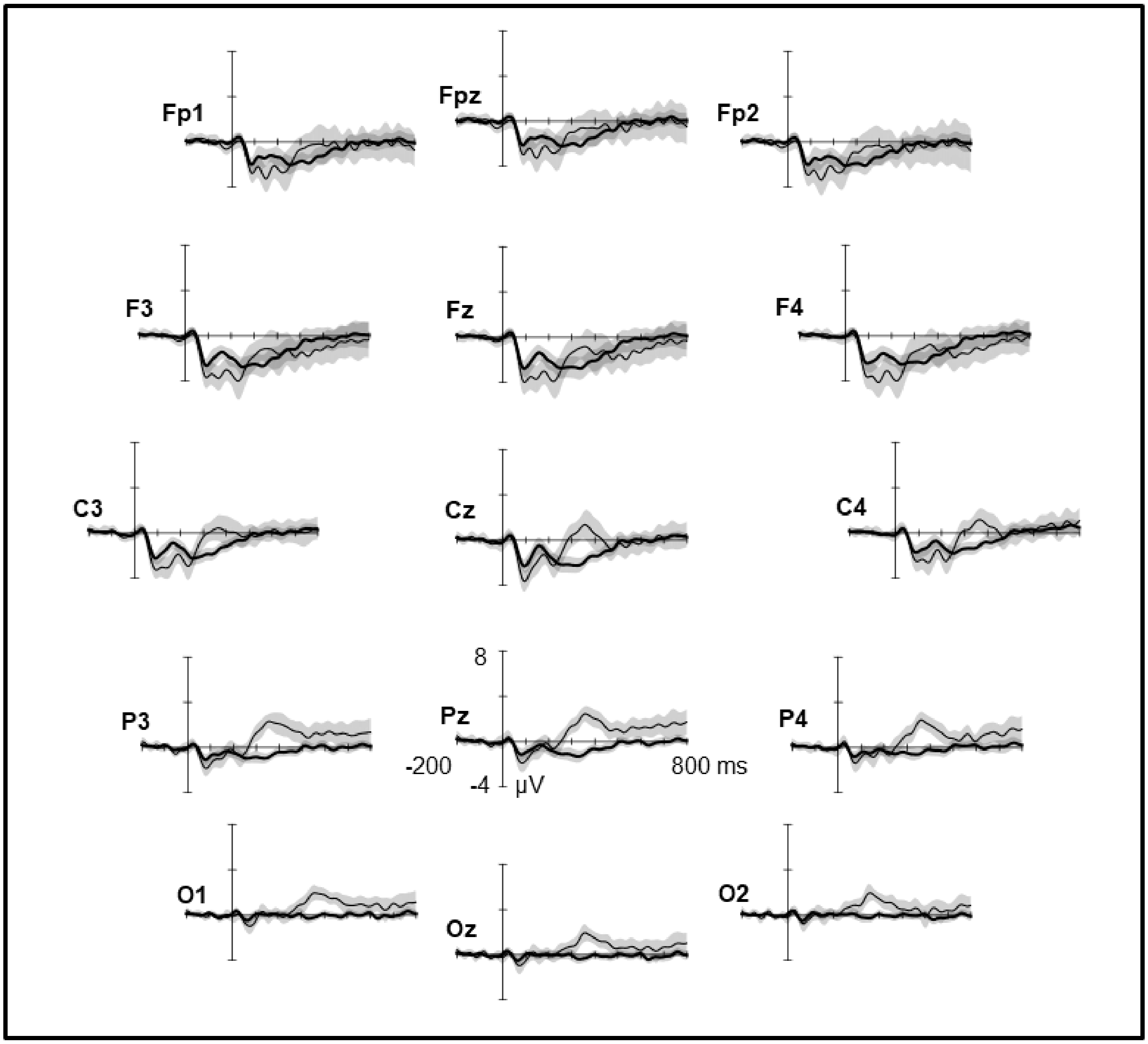


**Supplementary Figure 5: Experiment 2 - Wheeled hallway.** Distribution of grand-average (N=24) ERP waveforms at midline and medial electrode sites for the frequent (thick line) and infrequent (thin line) tones, recorded as participants performed the auditory oddball task while sitting in a wheelchair that is pushed along a hallway. These data are shown as in Supplementary Figure 1 and used to generate the P300 ERP difference waveforms shown in Figure 3.

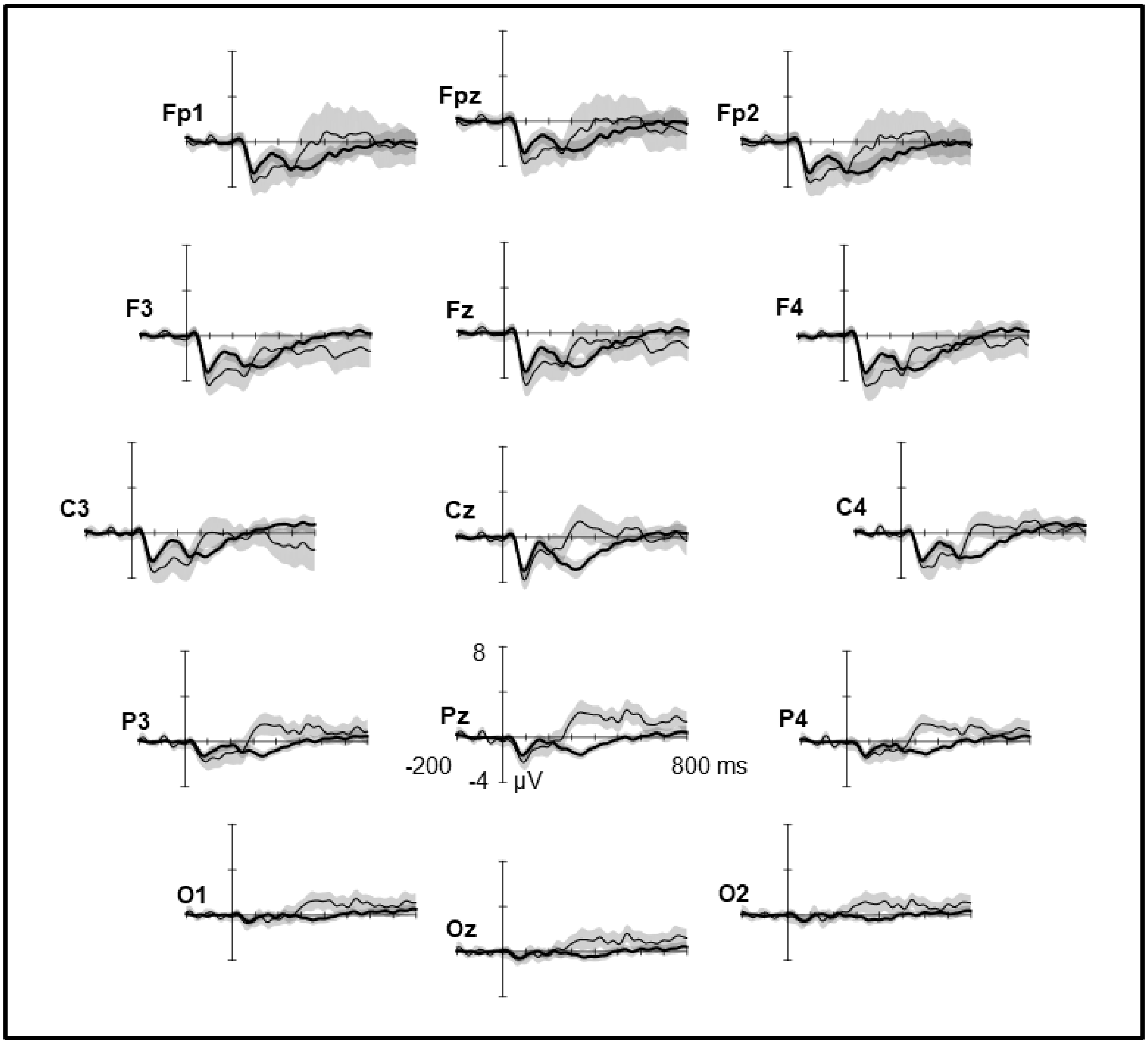


**Supplementary Figure 6: Experiment 2 - Walk hallway.** Distribution of grand-average (N=24) ERP waveforms at midline and medial electrode sites for the frequent (thick line) and infrequent (thin line) tones, recorded as participants performed the auditory oddball task while walking through a hallway. These data are shown as in Supplementary Figure 1 and used to generate the P300 ERP difference waveforms shown in Figure 3.

We also carried out additional examination of data quality in Experiment 2, analogous to those reported for Experiment 1. First, analysis revealed that the pre-stimulus baseline standard deviation was stable across conditions: mean (SD) voltage of .07 (.02), .07 (.02), .07 (.01) and .07 (.02) for standing, walk treadmill, wheeled hallway and walk hallway conditions respectively. Paired samples t-tests on the standard deviation of the baseline revealed no reliable difference across conditions (all Ps > .721). Second, an average of 6 (SD = 3) Independent Components (ICs) were rejected in each condition, and statistical analysis using paired samples t-tests revealed no significant difference in the number of ICs rejected across conditions (all Ps > .138). Finally, as in Experiment 1 the P300 was examined in a data-derived time window, defined as 2 standard deviations around the mean peak latency across all conditions (mean = 358 ms and SD = 40.8 ms, resulting in a 276-440 ms time window). Visual inspect of Figure 2 suggests that the P300 peak is very slightly delayed in the wheeled condition compared to all other conditions, but statistical analysis using paired-sample t-tests revealed no significant differences between any of the conditions (all P's > .052).

In Experiment 2 participants reported 98% of targets stimuli (SD = 1) in the standing condition, 95% of targets (SD = 3) in the walk hallway condition, 97% (SD = 2) in the walk treadmill condition, and 95% (SD = 2) in the wheeled condition. As noted in the main text, repeated measures ANOVA revealed a significant main effect of motion on task performance [$F(1, 23) = 32.547$, $p < .001$, $\eta^2 = .586$]. Task performance during standing was significantly higher than during both wheeled [$t(23) = 4.664$, $p < .001$, $d = 0.952$, $BF_{10}$ =

253.772] and hallway walking [$t(23) = 4.449$, $p < .001$, $d = 0.908$, $BF_{10} = 157.125$] conditions. There was, however, no significant difference in task performance between standing and treadmill walking conditions [$t(23) = 1.802$, $p = .085$]. Furthermore, task performance in the treadmill walking condition was significantly higher than in both the hallway walking [$t(23) = 2.511$, $p < .05$, $d = 0.513$, $BF_{10} = 2.780$] and wheeled [$t(23) = 2.136$, $p < .05$, $d = 0.436$, $BF_{10} = 1.451$] conditions. By contrast, there was no significant difference in performance between hallway walking and wheeled conditions [$t(23) = .394$, $p = .697$].

Additional correlational analysis comparing P300 amplitude and task performance revealed significant correlations in both the wheeled ($r_{23} = .468$, $p<.05$) and hallway walking ($r_{23} = .417$, $p<.05$) conditions. By contrast, no reliable correlations between task performance and P300 amplitude were found in either the standing ($r_{23} = .298$, $p = .157$) or treadmill ($r_{23} = .367$, $p=.078$) conditions, again most likely reflecting the fact that performance was very close to ceiling. Overall, the behavioural results suggest that task performance was influenced by the demands associated with displacement through space, rather than the act of walking itself.

***Experiment 3***

Supplementary Figures 7 to 10 show the ERP waveforms producing the P300 difference waveforms reported in Experiment 3. As for Experiment 2 we also provide additional information about trial numbers and data quality for Experiment 3. Average ERPs were derived from mean of 155 non-targets (range: 120-198) and 37 targets (range: 26-51) across

conditions and participants. For targets the means (and SDs) were: 37 (5) for wheeled static view, 35 (4) for wheeled dynamic view, 38 (4) for stationary static view and 38 (5) for stationary dynamic view. For non-targets the means (and SDs) were: 154 (16) for wheeled static view, 153 (18) for wheeled dynamic view, 155 (16) for stationary static view and 158 (13) for stationary dynamic view. As in Experiments 1 and 2, the number of trials remaining after artifact rejection was not significantly affected by recording condition. Analysis revealed that there was no significant effect of inertial stimulation on the amount of frequent [$F(1,23)$ =.799, $p$ = .381, $\eta^2$ = .034] or infrequent [$F(1,23)$ = 2.207, $p$ = .151, $\eta^2$ = .088] trials remaining after artifact rejection. Similarly, there was no significant effect of visual stimulation on the amount of frequent [$F(1,23)$ = .085 $p$ = .773, $\eta^2$ = .004], or infrequent [$F(1,23)$ = .637, $p$ = .433, $\eta^2$ = .027] trials remaining.

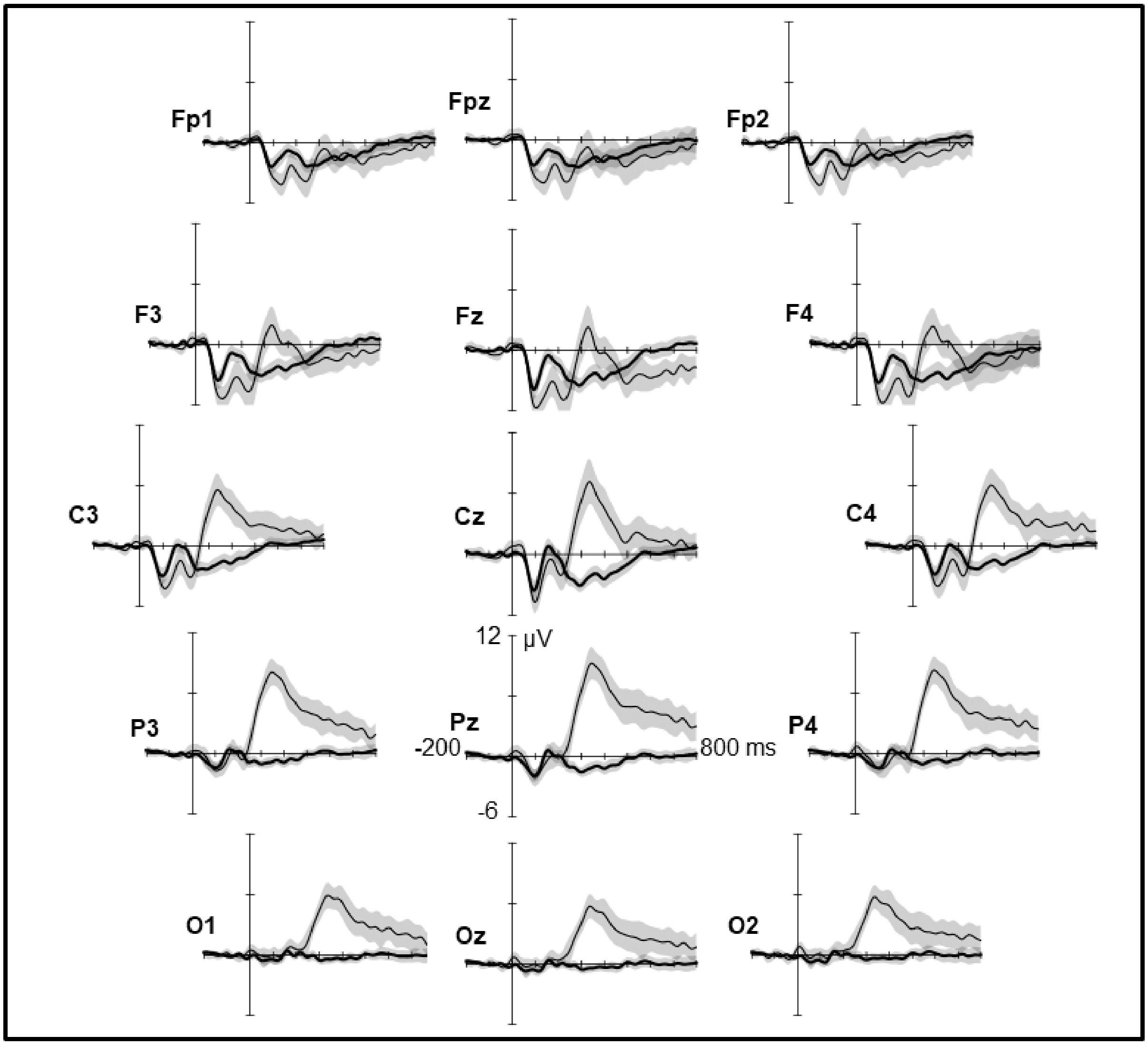


**Supplementary Figure 7: Experiment 3 - Stationary static view.** Distribution of grand-average (N=24) ERP waveforms at midline and medial electrode sites for the frequent (thick line) and infrequent (thin line) tones, recorded as participants performed the auditory oddball task while sitting facing a grey wall. Data are shown as in Supplementary Figure 1 and used to generate the P300 ERP difference waveforms shown in Figure 4.

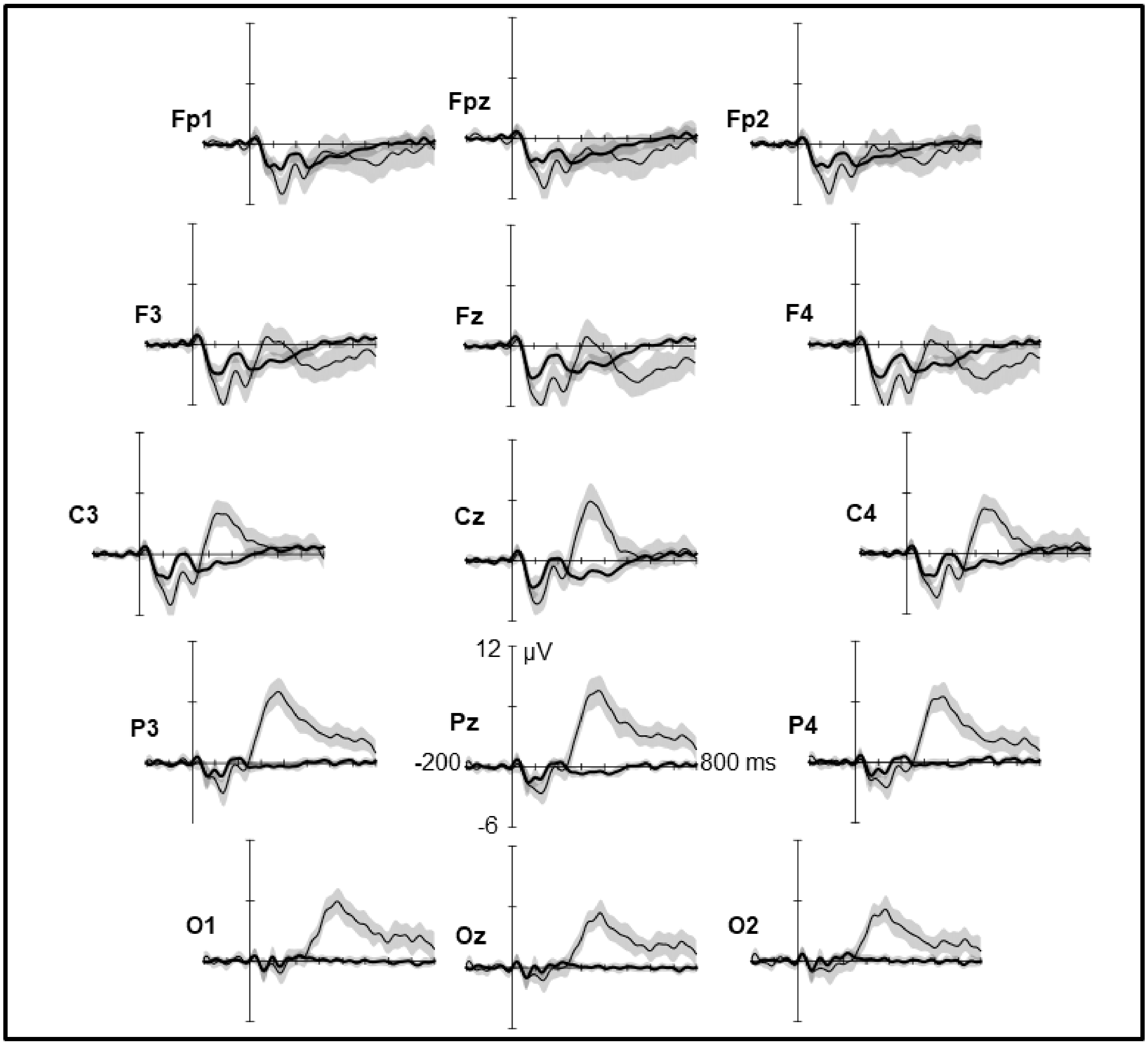


**Supplementary Figure 8: Experiment 3 - Wheeled static view.** Distribution of grand-average (N=24) ERP waveforms at midline and medial electrode sites for the frequent (thick line) and infrequent (thin line) tones, recorded as participants performed the auditory oddball task while wheeled along the hallway while watching a video of the grey wall. Data are shown as in Supplementary Figure 1 and used to generate the P300 ERP difference waveforms shown in Figure 4.

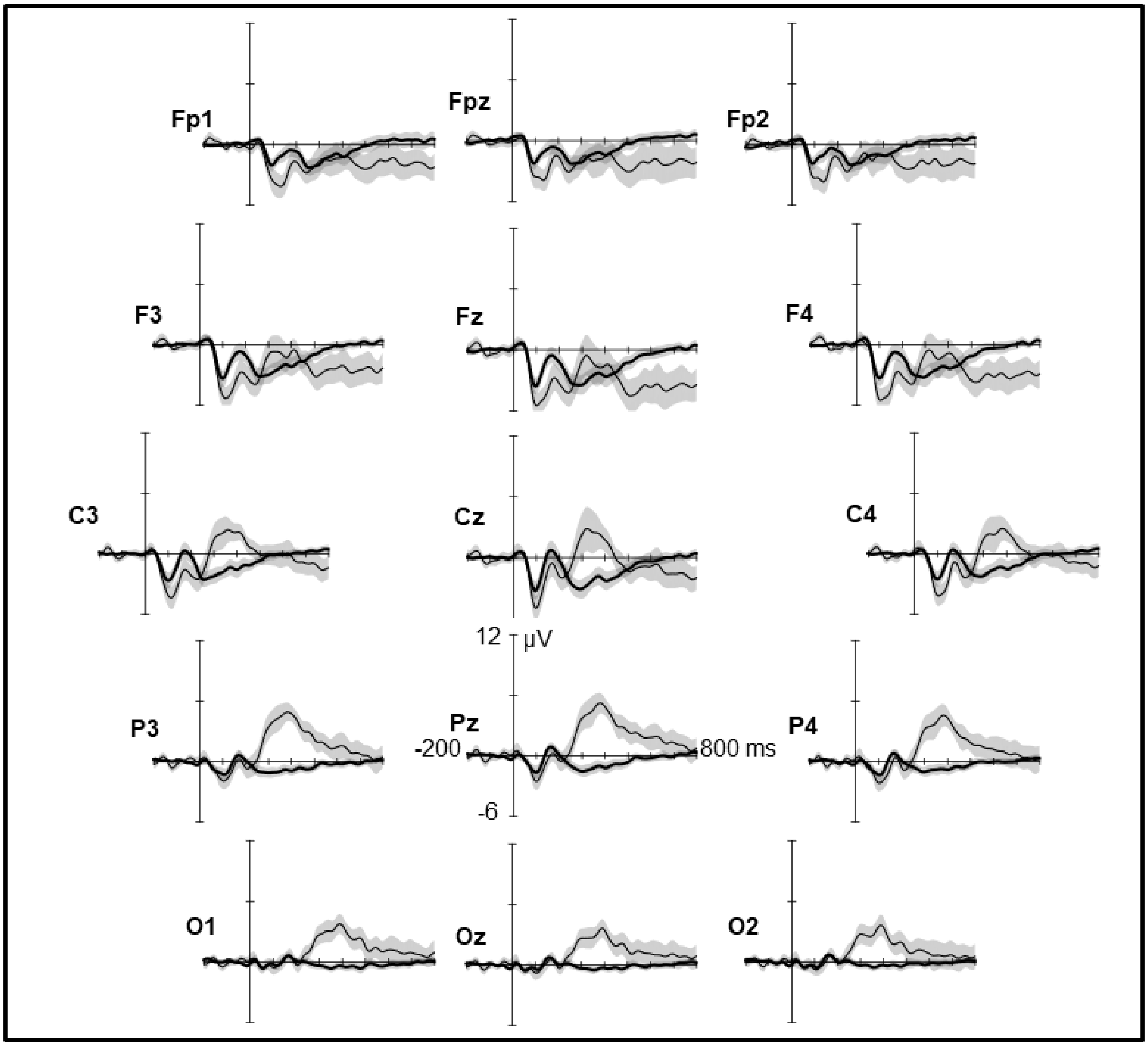


**Supplementary Figure 9: Experiment 3 - Stationary dynamic view.** Distribution of grand-average (N=24) ERP waveforms at midline and medial electrode sites for the frequent (thick line) and infrequent (thin line) tones, recorded as participants performed the auditory oddball task while sitting stationary and watching a video of traversal along the hallway. Data are shown as in Supplementary Figure 1 and used to generate the P300 ERP difference waveforms shown in Figure 4.

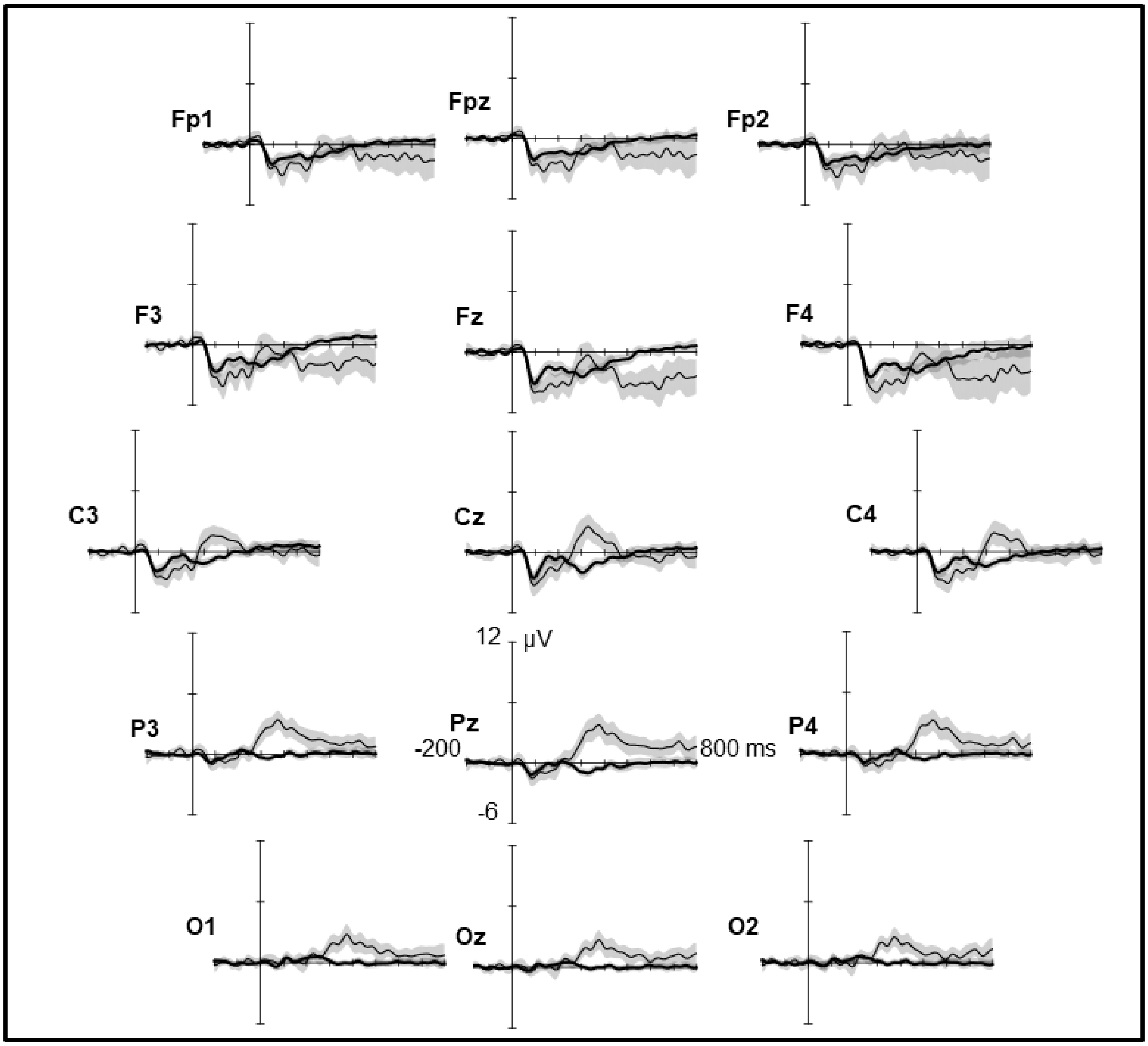


**Supplementary Figure 10: Experiment 3 - Wheeled dynamic view.** Distribution of grand-average (N=24) ERP waveforms at midline and medial electrode sites for the frequent (thick line) and infrequent (thin line) tones, recorded as participants performed the auditory oddball task while being wheeled along and freely viewing the hallway. Data are shown as in Supplementary Figure 1 and used to generate the P300 ERP difference waveforms shown in Figure 4.

In contrast to Experiments 1 and 2, assessment of data quality revealed that the pre-stimulus baseline did exhibit small differences in Experiment 3. Mean micro-voltage of the baseline period standard deviation was .07 (.02), .07 (.02), .08 (.02), and .08 (.02) for the wheeled dynamic view, wheeled static view, stationary dynamic view, and stationary static view conditions respectively. Paired samples t-tests revealed that the wheeled dynamic view condition had significantly lower baseline standard deviation than both the stationary static view [$t(23) = 2.758$, $p < .05$], and stationary dynamic view [$t(23) = 2.643$, $p < .05$] conditions. The significant difference in the baseline reflects low levels of variability in the wheeled dynamic view condition (visible in Figure 3). Importantly, however, the difference in baseline standard deviations cannot account for the differences in the magnitude of the P300 effect between conditions that are reported in the main text (and further confirmed by pairwise comparisons of the P300 effect reported below).

A significant interaction between inertial stimulation and visual stimulation was found on the number of artifactual ICs rejected [$F(1,23) = 4.979$, $p < .05$, $\eta^2 = .178$]. Analysis revealed that the number of ICs rejected during the pre-processing of wheeled dynamic view condition (mean = 6, SD = 2) was significantly higher than in both the stationary static view [mean = 5, SD = 2: $t(23) = 2.186$, $p < .05$], and the stationary dynamic view [mean = 4, SD = 2: $t(23) = 3.006$, $p < .01$], but also relatively higher than in the wheeled static view [mean = 5, SD = 2: $t(23) = 2.013$, $p = .056$]. Importantly, however, there were no reliable differences in the number of artifactual ICs found between the stationary static view, stationary dynamic view and wheeled static view conditions. Hence, as for the baseline, differences in

the number of artifactual ICs cannot account for the critical pattern of P300 results. Finally, the P300 was once again examined in a data-derived time window, defined as 2 standard deviations around the mean peak latency across all conditions (mean = 363 ms and SD = 41.4 ms, resulting in a 280-446 ms time window). Statistical analysis using paired-sample t-tests revealed no significant differences in peak latency between any of the conditions (all P's > 0.077).

Given the evidence of differences in baseline standard deviation and artifactual ICs outlined above, we carried out additional post-hoc analyses to directly compare the magnitude of the P300 effect across each pair of conditions. Consistent with the results reported in Figure 3, analysis revealed that the P300 effect was largest when participants sat facing a grey wall, and significantly larger than when sat stationary watching a video of traversal [$t(23) = 4.901$, $p < .001$, $d = 1.000$, $BF_{10} = 431.020$]; when wheeled along watching a video of the grey wall [$t(23) = 3.255$, $p < .01$, $d = .664$, $BF_{10} = 11.853$]; and when wheeled along the hallway while freely viewing the traversal [$t(23) = 7.105$, $p < .001$, $d = 1.450$, $BF_{10} = 53036.052$]. The smaller P300 effect seen when participants were wheeled along the hallway watching a video of the grey wall was still significantly larger than the P300 effect seen when participants were sat stationary watching a video of the hallway traversal [$t(23) = 2.265$, $p < .05$, $d = .462$, $BF_{10} = 1.800$] and when wheeled along the hallway while freely viewing the traversal [$t(23) = 5.803$, $p < .001$, $d = 1.191$, $BF_{10} = 3429.427$]. Finally, the smaller P300 effect observed when participants were sat stationary watching a video of the hallway traversal was still larger than the P300 effect seen when participants were wheeled along

the hallway while freely viewing the traversal ($t(23) = 5.098$, $p < .001$, $d = 1.041$, $BF_{10}$ = 668.965), the condition associated with the smallest P300 effect compared to all other conditions. Taken together, these analyses provide clear evidence that the differences in magnitude of the P300 effect reported in Experiment 3 do not reflect simply reflect changes in baseline stability or artifact rejection levels, but instead reveal differences in the capture of attention consistent with our experimental manipulations.

Analysis of the behavioural data from Experiment 3 revealed that vestibular information did not yield a significant main effect on task performance [$F(1,23) = 1.835$, $p = .189$, $\eta^2 = .074$], but visual information did [$F(1,23) = 30.918$, $p = .000$, $\eta^2 = .573$], as highlighted in the main text. Follow up paired-samples t-tests revealed that task performance was significantly higher during the stationary static view condition (mean = 98%, SD = 1) than both the wheeled dynamic view [mean = 95%, SD = 2; $t(23) = 4.833$, $p < .001$, $d = 0.987$] and stationary dynamic view conditions [mean = 95.34%, SD = 2.55; $t(23) = 4.703$, $p < .001$, $d = 0.960$]. While task performance was not significantly different between stationary static view and wheeled static view conditions [mean = 97%, SD = 3; $t(23) = 1.639$, $p = .115$], task performance in the wheeled static view condition was significantly higher than in the stationary dynamic view [$t(23) = 2.769$, $p < .05$, $d = 0.565$] and the wheeled dynamic view conditions [$t(23) = 2.225$, $p < .05$, $d = 0.454$]. There was no significant difference in task performance between wheeled dynamic view and stationary dynamic view conditions [$t(23)= .094$, $p = .926$].

As in Experiments 1 and 2 we also carried out correlational analysis, revealing significant correlations between P300 amplitudes and task performance in the stationary dynamic view ($r_{23} = .418$, $p < .05$) and the wheeled dynamic view ($r_{23} = .406$, $p < .05$) conditions. By contrast, for the stationary static view ($r_{23} = .333$, $p = .112$) and the wheeled static view ($r_{23} = .383$, $p = .065$) conditions, where performance was closest to ceiling, the correlational analysis did not reach significance. Overall, the behavioural results suggest that task performance was influenced most by the additional demands associated with an increase of visual flow of information, consistent with the presence of larger decreases in the magnitude of the P300 effect for visual than inertial stimulation.
